# Supplementary material for: Charting differentially methylated regions in cancer with Rocker-meth
Source: Commun Biol. 2021 Nov 2;4:1249. doi: 10.1038/s42003-021-02761-3 (PMC8563962; doi:10.1038/s42003-021-02761-3)
Supplement: Supplementary file 2 — Supplementary Information [file 42003_2021_2761_MOESM2_ESM.pdf]

Supplementary Information

Charting differentially methylated regions in cancer with Rocker-meth

Matteo Benelli[1*§], Gian Marco Franceschini[2§], Alberto Magi[3], Dario Romagnoli[1], Chiara Biagioni[1,4], Ilenia Migliaccio[5], Luca Malorni[4,5], Francesca Demichelis[2,6]

1 Bioinformatics Unit, Hospital of Prato, Prato, Italy;

2 Department of Cellular, Computational and Integrative Biology, University of Trento, Italy;

3 Department of Information Engineering, University of Florence, Florence, Italy;

4 “Sandro Pitigliani” Medical Oncology Department, Hospital of Prato, Prato, Italy;

5 “Sandro Pitigliani” Translational Research Unit, Hospital of Prato, Prato, Italy.

6 Institute for Computational Biomedicine, Weill Cornell Medical College, New York, NY, USA.

§ These authors equally contributed to this work.

* To whom correspondence should be addressed. Email: matteo.benelli@uslcentro.toscana.it or f.demichelis@unitn.it

Supplementary Note 1

Parameter study of the HMM based segmentation algorithm

To set Rocker-meth parameters, prior to the comparative analysis with other tools (see next section), we performed a comprehensive simulation study only on synthetic RRBS datasets (parameters training dataset) to evaluate the performance of the segmentation algorithm of Rocker-meth while varying the values of its parameters. These analyses allowed us to identify the optimal configuration of these parameters. We first carried out a comprehensive simulation study on the synthetic RRBS datasets to evaluate the performance of the segmentation algorithm of Rocker-meth while varying the values of its parameters *Dnorm*, *p*, *F* and *mu*. RRBS data display a CpG genomic coverage typically higher than array-based technologies and lower than WGBS. In our parameterization, *mu* is the mean of state 1 (μ_1 hypo-methylation). The mean of state 3 (μ_3) is defined as *1-mu*, while the mean of not differentially methylated state is set to 0.5.The following values of the parameters were tested:

- *Dnorm*: 10, 10^2, 10^3, 10^4, 10^5, 10^6;
- *F*: 0.1 to 0.9 by 0.1;
- *p*: 0.01, 0.02, 0.03, 0.04, 0.05, 0.06, 0.07, 0.08, 0.09, 0.10, 0.15, 0.25, 0.35, 0.45;
- *mu*: 0.05, 0.10. 0.15, 0.20, 0.25, 0.30, 0.35.

Results of the analyses are summarized in Supplementary Figure S1. The simulation study shows that our method is very sensitive to *F* and *mu* (**Supplementary Figure 1a,b**) whereas is only mildly affected by *p* and *Dnorm* (**Supplementary Figure 1c,d**). For parameter *F*, this result is in line with the results obtained in our previous works on the implementation of HMM segmentation algorithms for the analysis of somatic copy number from array-CGH and WES [74–76] and regions of homozygosity [39]. As for parameter *mu* (mean level of hypo-methylated state; 1-mu is for hyper-methylated state), this is expected considering that the synthetic datasets are characterized by different levels of noise that result in different distributions of the AUCs of differentially methylated states. To study the interdependence between *F* and *mu*, we studied their association across the different classes of synthetic datasets. Results are summarized in **Supplementary Figure 2**. As expected, the combinations of *F* and *mu* values giving the best performance in terms of F1 vary across the different synthetic datasets: for

classes 1 to 3 we obtained *mu= [0.15, 0.30]* and *F= [0.3, 0.6]*, while for classes 4 and 5 we obtained *mu= [0.2, 0.30]* and *F=[0.4, 0,6]*. Overall, these data indicate that a reasonable choice is *mu=0.25* and *F=0.4,* that we set as default values in our method). Indeed, as already reported in our previous works [39,74,75], *F* should be kept as low as possible to avoid over-segmentation. We then further investigated the dependence of our model from the parameters *Dnorm* and *p*. To do that, we set *F* and *mu* to default values and studied the association between *Dnorm* and *p* across the different synthetic datasets (class 1 to 5). The results reported in Supplementary Figure S3 suggest that the best performances in terms of F1 are obtained for higher values of *Dnorm* and lower values of *p*. For this reason, default values for *p* and *Dnorm* were set to *p=0.05* and *Dnorm = 1e+5*. All the data related to the HMM parameter study is available at http://doi.org/10.5281/zenodo.2586588. Importantly, we kept those parameters fixed throughout the analysis of all other synthetic and real data, independently of the experimental platforms, obtaining consistently good performance compared to the other tested tools (**Figure 1c**).

Minimum number of sites for defining reliable DMRs

To define the threshold of the number of sites for nominating reliable DMRs, we studied the distribution of the number of sites supporting false-positive segments detected by Rocker-meth in the synthetic datasets. Overall, we observed that false-positive segments tend to be supported by a lower number of sites for datasets characterized by higher noise (classes 4 and 5 vs 1-3), as expected. For HM450, we obtained no false-positive segment supported by more than 5 sites and the same value also corresponded to the fourth quartile of the distribution for WGBS data. We therefore adopted nsites = 6 as a reasonable threshold for calling reliable DMRs in both HM450 and BS-seq real data. The results of this analysis are reported in **Supplementary Figure 3**.

Evaluation of Rocker-meth performance in case of small set of control samples

We evaluated the performance of Rocker-meth in the analysis of unbalanced datasets (few normal samples and many tumor samples). To do that we applied Rocker-meth to subsets of TCGA-PRAD samples, by randomly selecting n=5 and n=10 normal samples from a total of 50

normal samples (entire dataset). To study the effect of lower normal sample numbers in the estimation of AUCs, we averaged the AUC values obtained from 20 random sampling and compared with original values. As reported in Figure S11, we observed a high correlation between original AUC values and AUC obtained from the sub-selection of n=5 (**Supplementary Figure 36a**) and n=10 (**Supplementary Figure 36b**) normal samples. As expected, Pearson's correlation coefficient R was higher for n=10 vs all than n=5 vs all. As reported in the bottom panel of the figure, we observed that the AUC differences were more marked for original AUC values vs n=5 analysis (**Supplementary Figure 36c**) than n=10 (**Supplementary Figure 36d**) and for both cases taking values in the range 0.3-0.7. To evaluate how these discordances in terms of AUC estimations impact the DMR calls, we applied Rocker-meth to all these cases and studied the overlap between original DMRs and DMRs obtained from the two down-sampling strategies. Here we define Recall as the fraction of original DMRs detected by normal down-sampling analyses. Results are reported in **Supplementary Figure 37** and show detection of 88% and 90% (median values) of original hyper DMRs (left) and of 85% and 86% of original hypo DMRs (right) for n=5 and n=10 sampling, respectively.

We also evaluated the performance of Rocker-meth including all the detected DMRs and not only those with FDR < 0.05 (default), as Rocker-meth's DMR statistics depends on the number of cases. As reported in **Supplementary Figure 38** we observed a marked increase in recall obtaining a median value of 0.93 and 0.97 for hyper DMRs in case of n=5 and n=10 normal samples respectively and recall = 0.92 and 0.97 for hypo DMRs in case of n=5 and n=10 normal samples respectively. To evaluate the effect of imbalanced analysis on DMRs in terms of AUC scores only (i.e., not considering the contribution of segmentation), we studied the correlation between the AUC scores obtained in the down-sampling study versus the original AUC scores in the DMRs detected by Rocker-meth in the original dataset. The results are reported in **Supplementary Figure 39** and show that AUC scores obtained using both n=5 and n=10 controls very well recapitulate original values. We observed that AUC scores with less control samples tends to be systematically lower in case of hyper DMRs or higher in case of hypo-DMRs and hypo-blocks, indicating that a higher number of control samples positively affects the AUC estimation, as expected.

Supplementary Figures

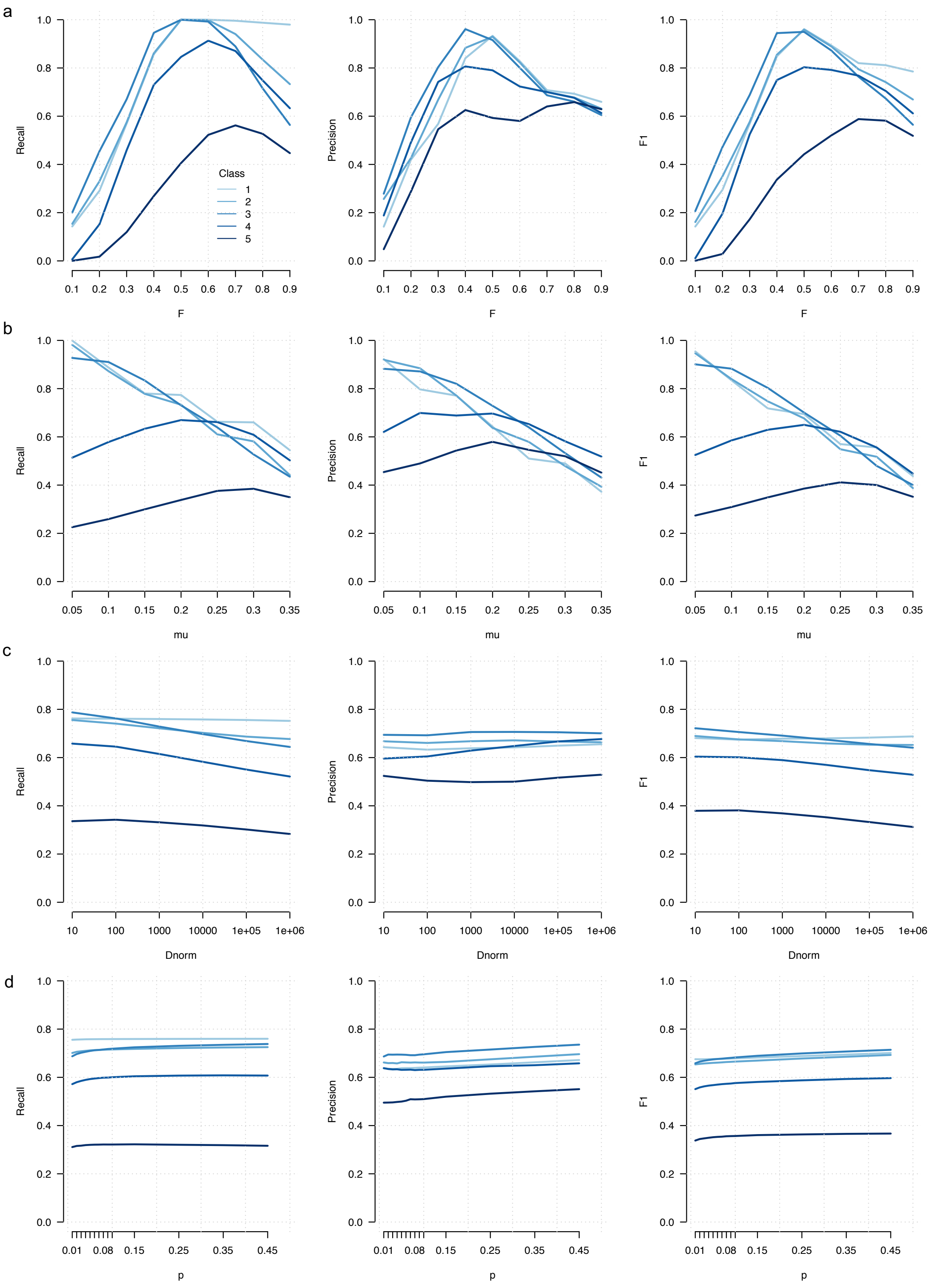

Supplementary Figure 1. Recall, precision and F1 statistical measures for different settings of the model's parameters (a) F, (b) mu, (c) Dnorm and (d) p in the five classes of synthetic RRBS datasets (blue lines).

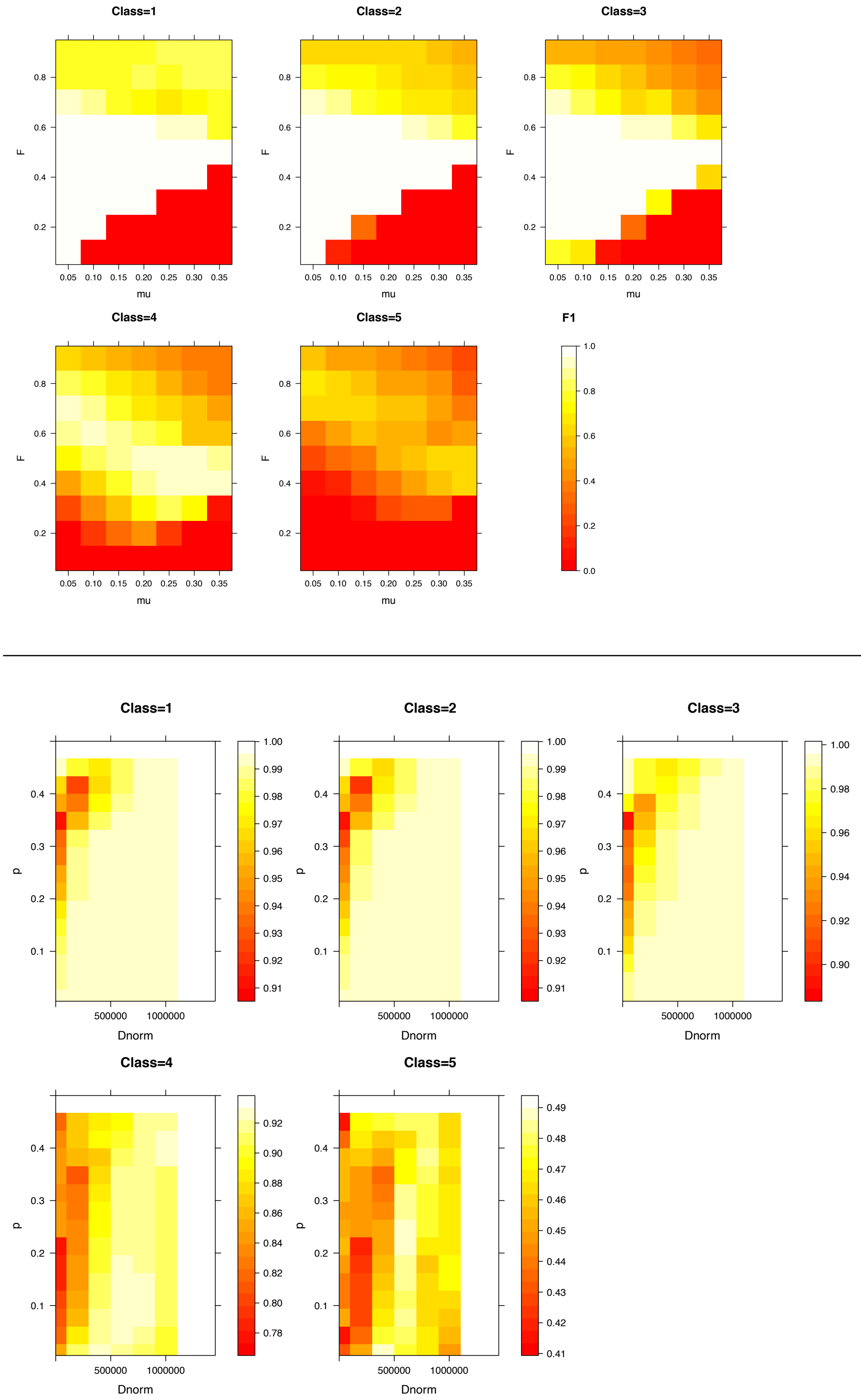

Supplementary Figure 2. Top panel: Level plots of F1 measure (different color levels) for the different settings of F and mu for the five RRBS synthetic datasets. Bottom panel: Level plots of F1 measure (different color levels) for the different settings of p and Dnorm the five RRBS synthetic datasets. Based on the analysis reported in Figure R5.2, F and mu were set to 0.4 and 0.25, respectively.

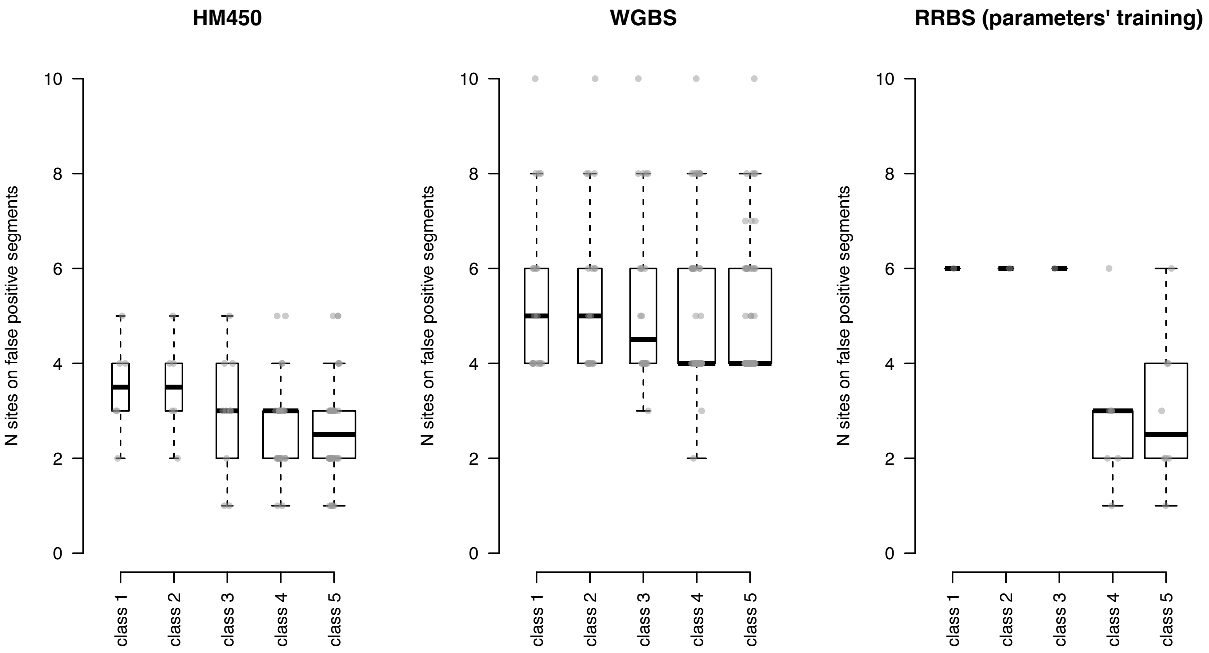

Supplementary Figure 3. Box plots of the distribution of the number of false positive segments detected by Rocker-meth in the HM450, RRBS and WGBS synthetic datasets. For high signal-to-noise ratio RRBS datasets (classes 1/2/3) Rocker-meth identified one false positive segment.

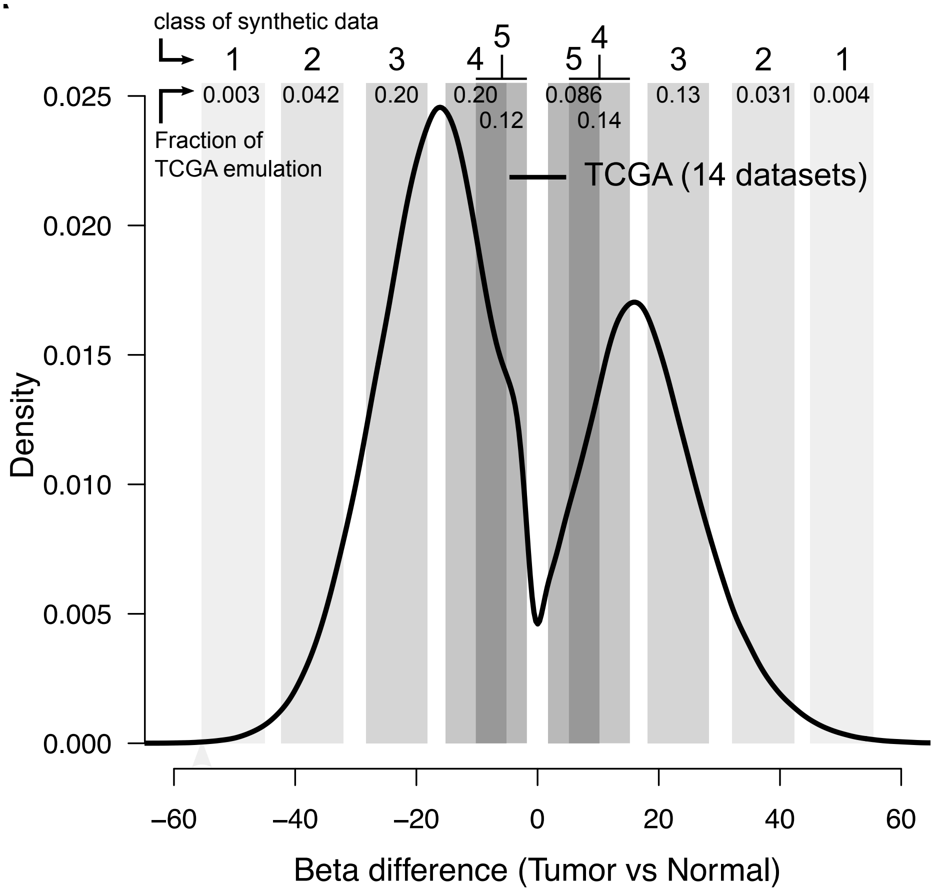

Supplementary Figure 4. Distribution of the differences of per site percentage of methylation (beta) between tumor and normal samples for all differentially methylated sites across 14 tumor types (TCGA datasets). Overlaid grey bars refer to the range of beta difference classes as defined on synthetic datasets and termed from class 1 (highest signal-to-noise ratio) to class 5 (lowest signal-to-noise ratio). The fraction of emulated TCGA data is reported at the top of each bar.

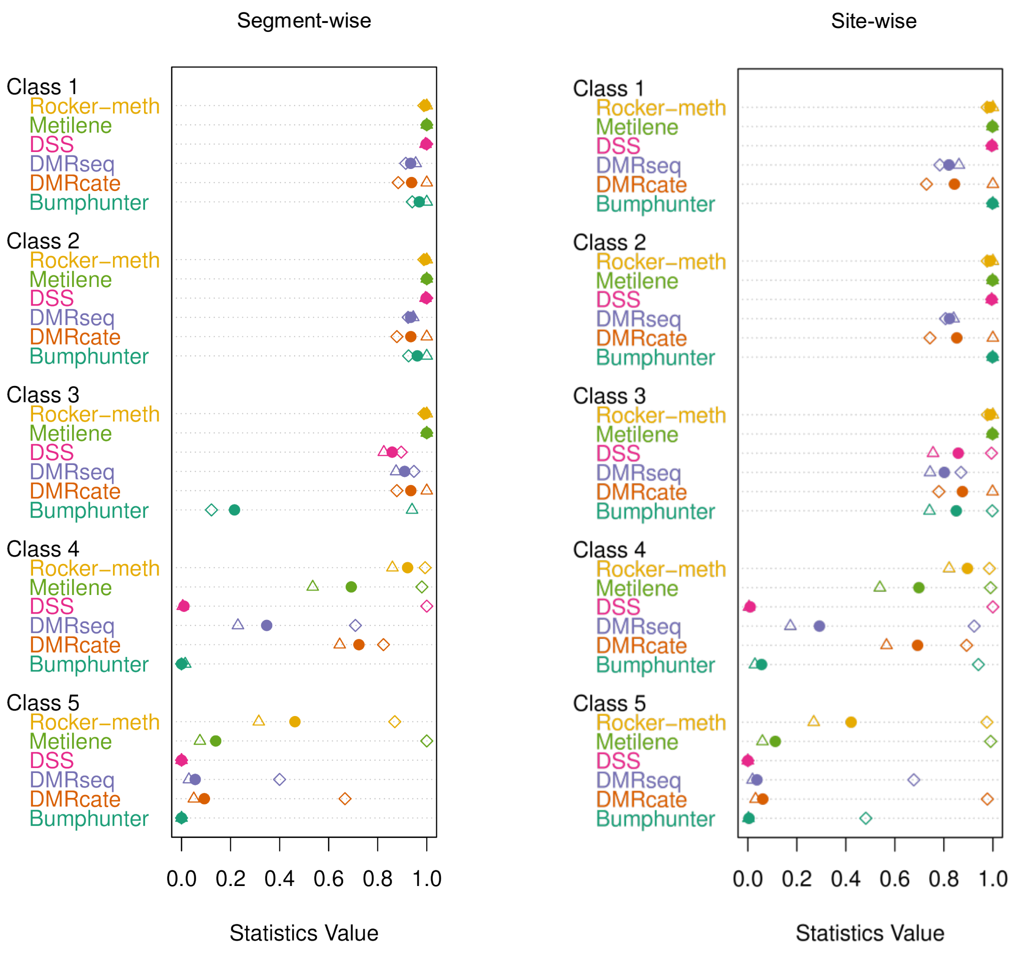

Supplementary Figure 5. Dot chart summarizing segment wise (left) and site-wise (right) precision (diamonds), recall (triangles) and F1 (filled circles) measures for Rocker-meth and other state-of-the-art methods in the artificial RRBS datasets used for parameter training.

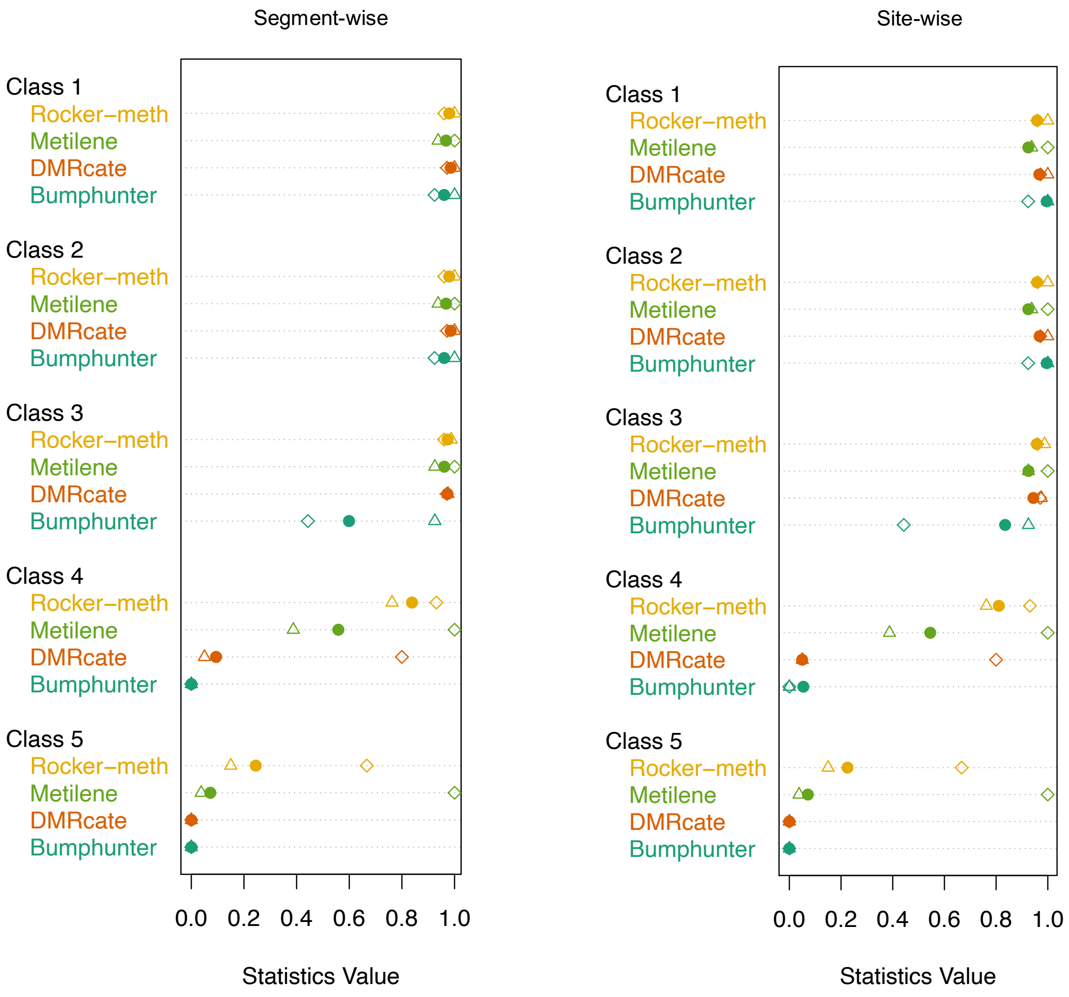

Supplementary Figure 6. Dot chart summarizing segment wise (left) and site-wise (right) precision (diamonds), recall (triangles) and F1 measures (filled circles) for Rocker-meth and other state-of-the-art methods in the artificial 450K datasets.

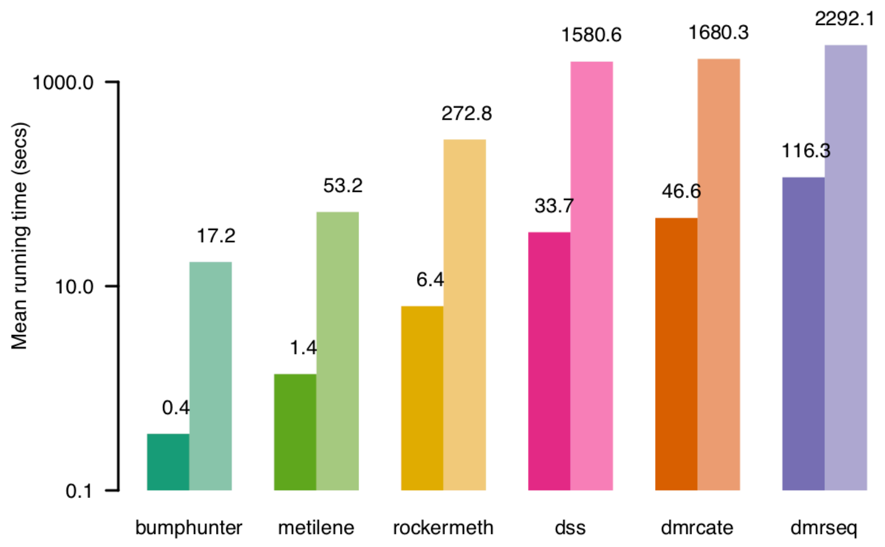

Supplementary Figure 7. Mean running times (secs/CPU) for Rocker-meth and the other state-of-the-art methods considered in this study. Darker color refers to RRBS datasets, lighter color refers to WGBS datasets.

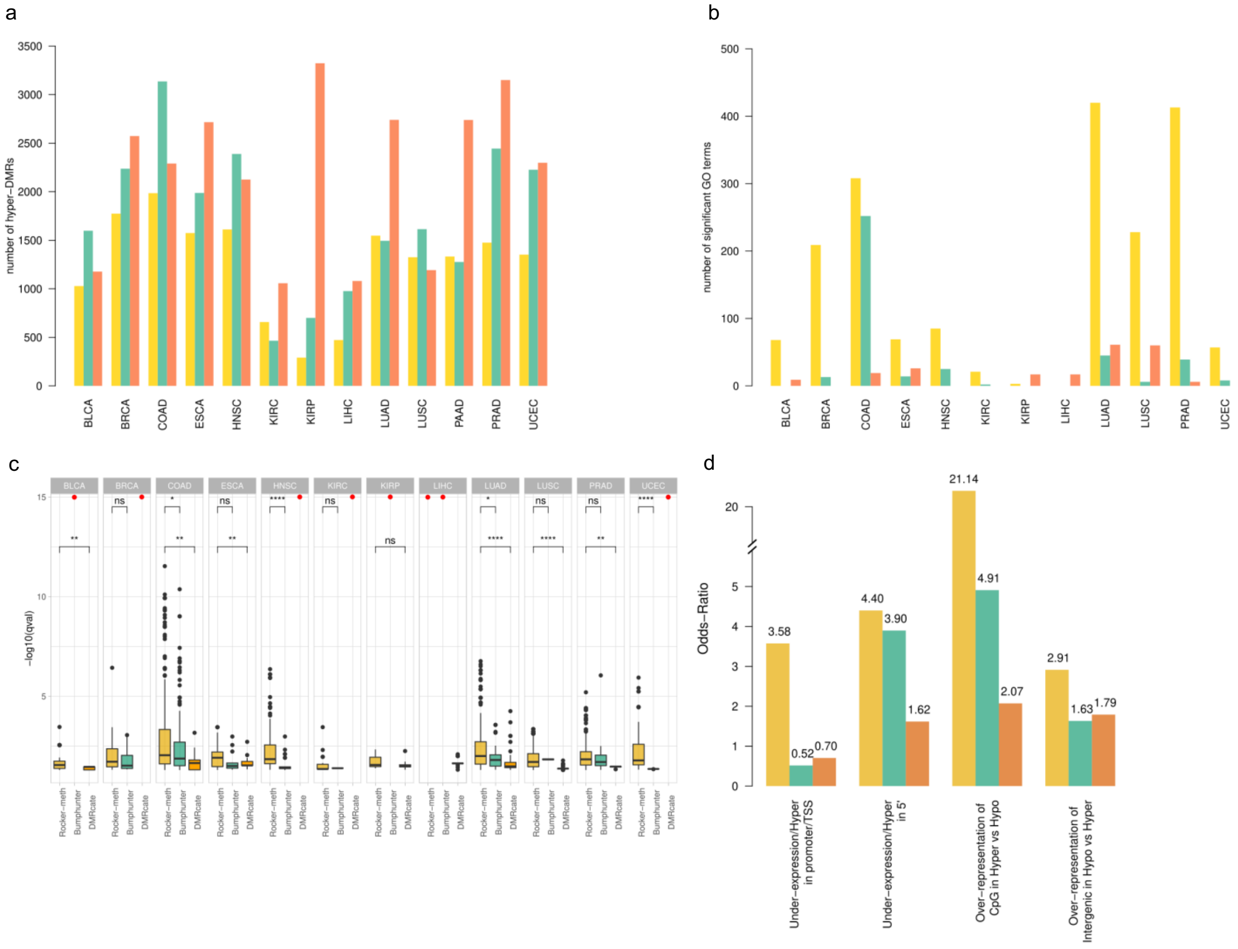

Supplementary Figure 8. a) Bar plots of the number of hyper-DMRs detected by Rocker-meth (yellow), Bumphunter (green), and DMRcate (orange) across the different tumor types. b) Bar plots of the number of significant (FDR<0.05) Gene Ontology (GO) terms resulting from the analysis of hyper-DMRs in promoter-TSS, 5' UTR or first intron of under-expressed genes. c) Box plots showing the distribution of -log10 of FDR from the clusterProfiler analysis on the set of Gene Ontology (GO) terms using events detected by Rocker-meth, Bumphunter, and DMRcate across the different tumor types. Red points indicate when no significant term has been found. d) Bar plots show the ability of Rocker-meth, Bumphunter, and DMRcate in recapitulating well known DNA methylation features in human cancers, estimated by Odds-ratio.

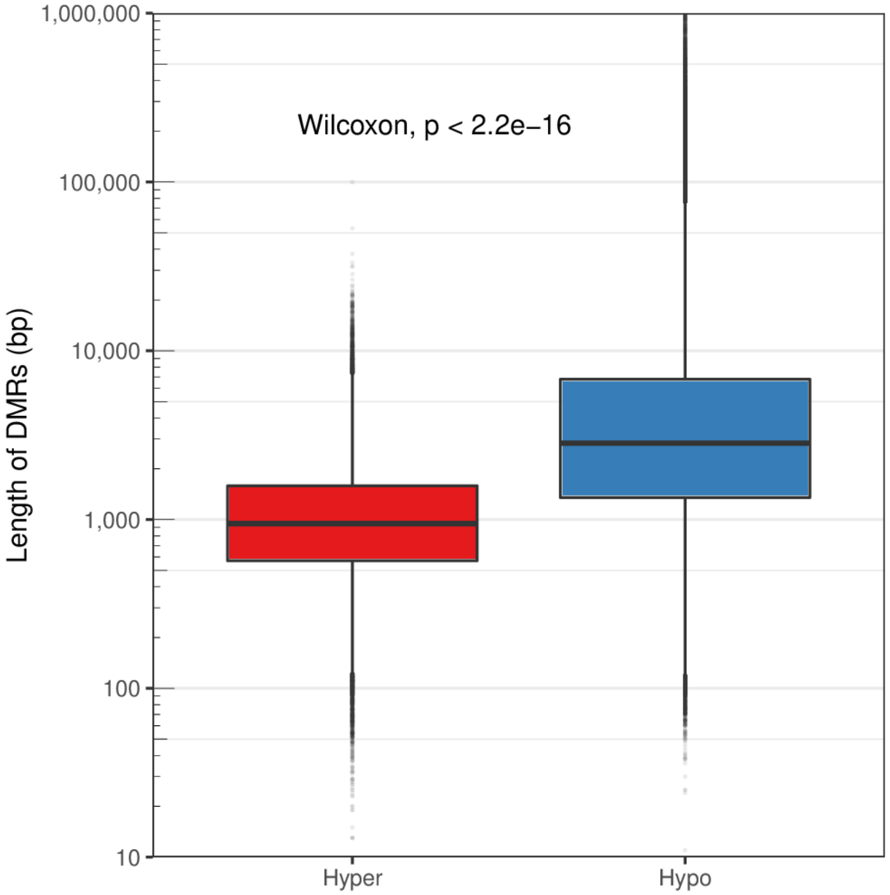

Supplementary Figure 9. Box plots of the distribution of the length of gain-of-methylation (hyper) and loss-of-methylation (hypo) DMRs across 6 WGBS datasets. P-value of the difference is estimated by Wilcoxon-Mann-Whitney test.

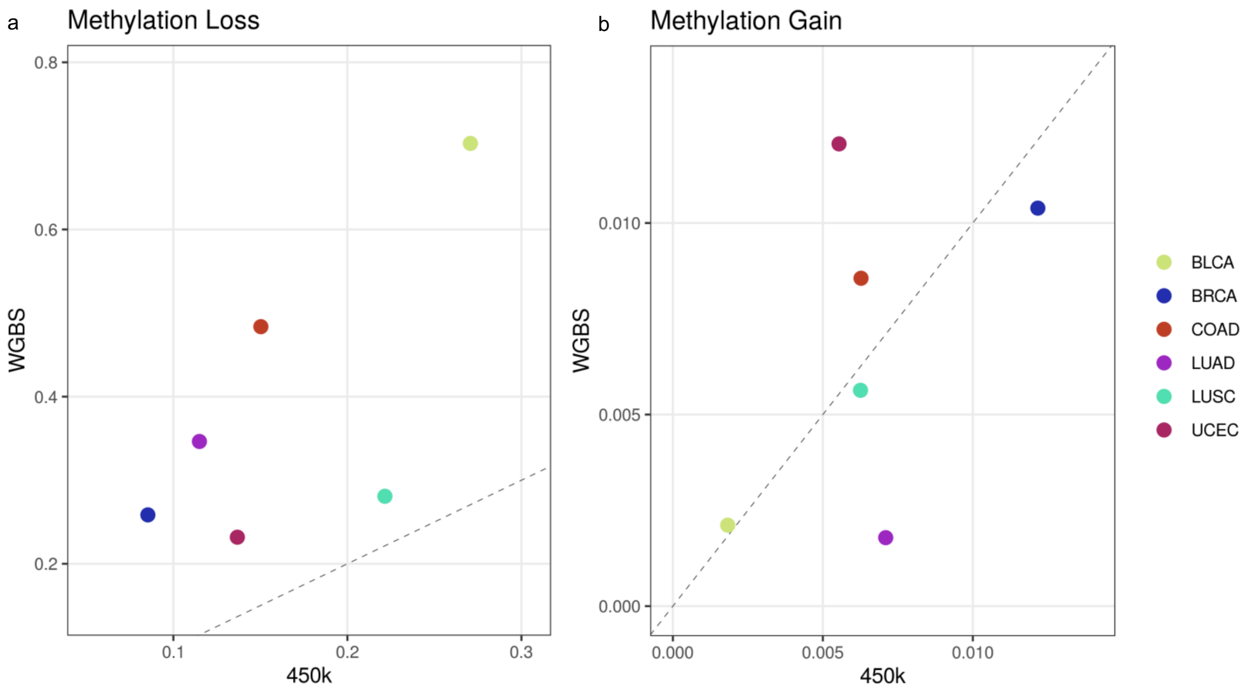

Supplementary Figure 10. Scatter-plot of DMR-burden for loss-of-methylation (a) and gain-of-methylation (b) events detected by Rocker-meth in WGBS (y-axis) vs HM450 (x-axis).

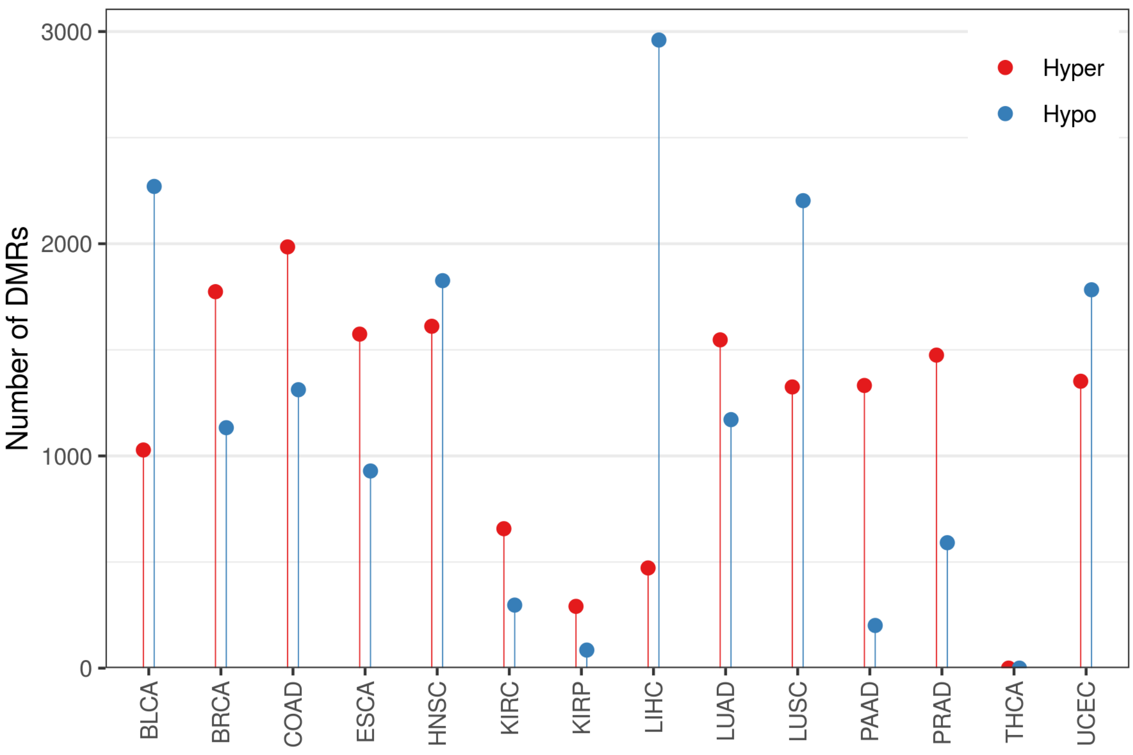

Supplementary Figure 11. Dot plot of number of DMRs across 14 HM450k TCGA datasets.

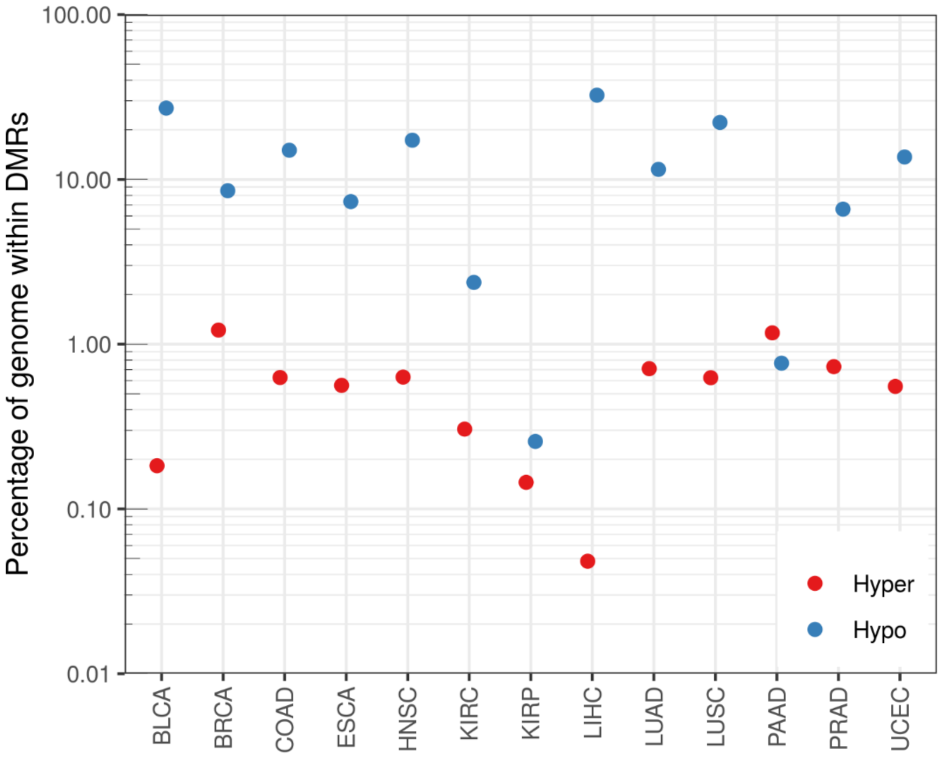

Supplementary Figure 12. Distribution of DMR burden (i.e, fraction of cancer genome within DMRs) across 13 HM450k TCGA datasets (THCA excluded).

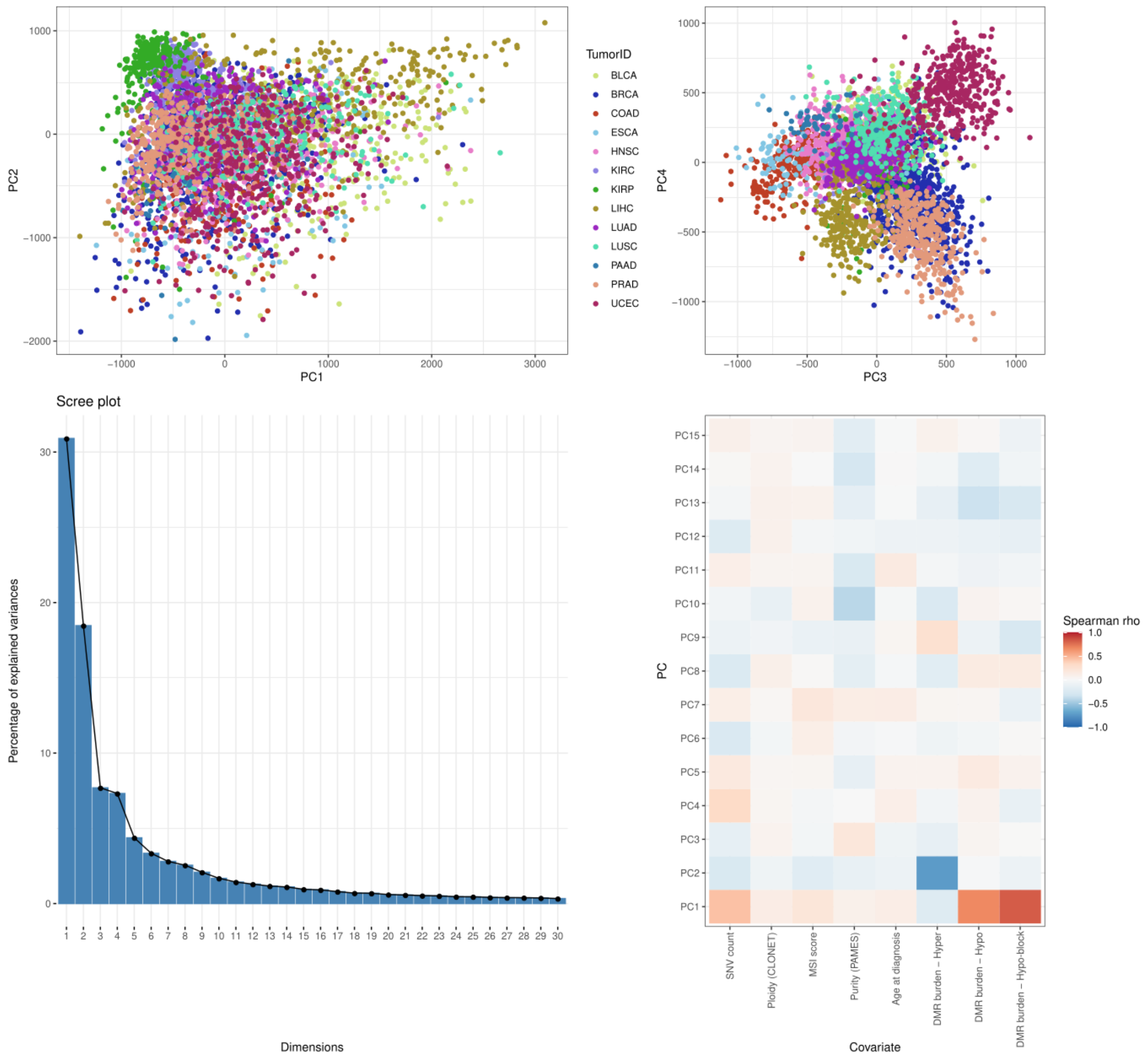

Supplementary Figure 13. Principal component analysis based on Beta difference in DMR consensus set. Top row: visualization of PC1/PC2 (left) and PC3/PC4 (right). Dots are single samples, and their average beta difference has been computed against the median of matched normal samples. Bottom row: diagnostic scree plot of top 30 principal components (left) and Spearman correlation coefficients between the top 15 principal components and several covariates, including tumor purity, age at diagnosis, SNV count and MSI score.

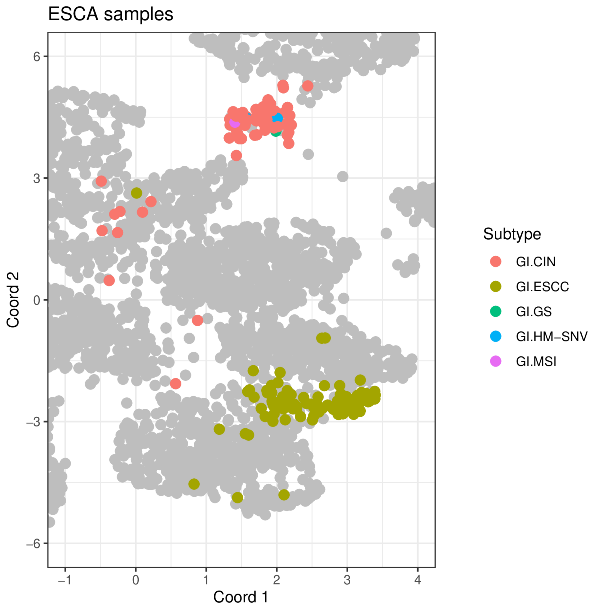

Supplementary Figure 14. UMAP projection for TCGA-ESCA samples by subtypes as in Figure 3D. Grey dots represent samples of other tumor types.

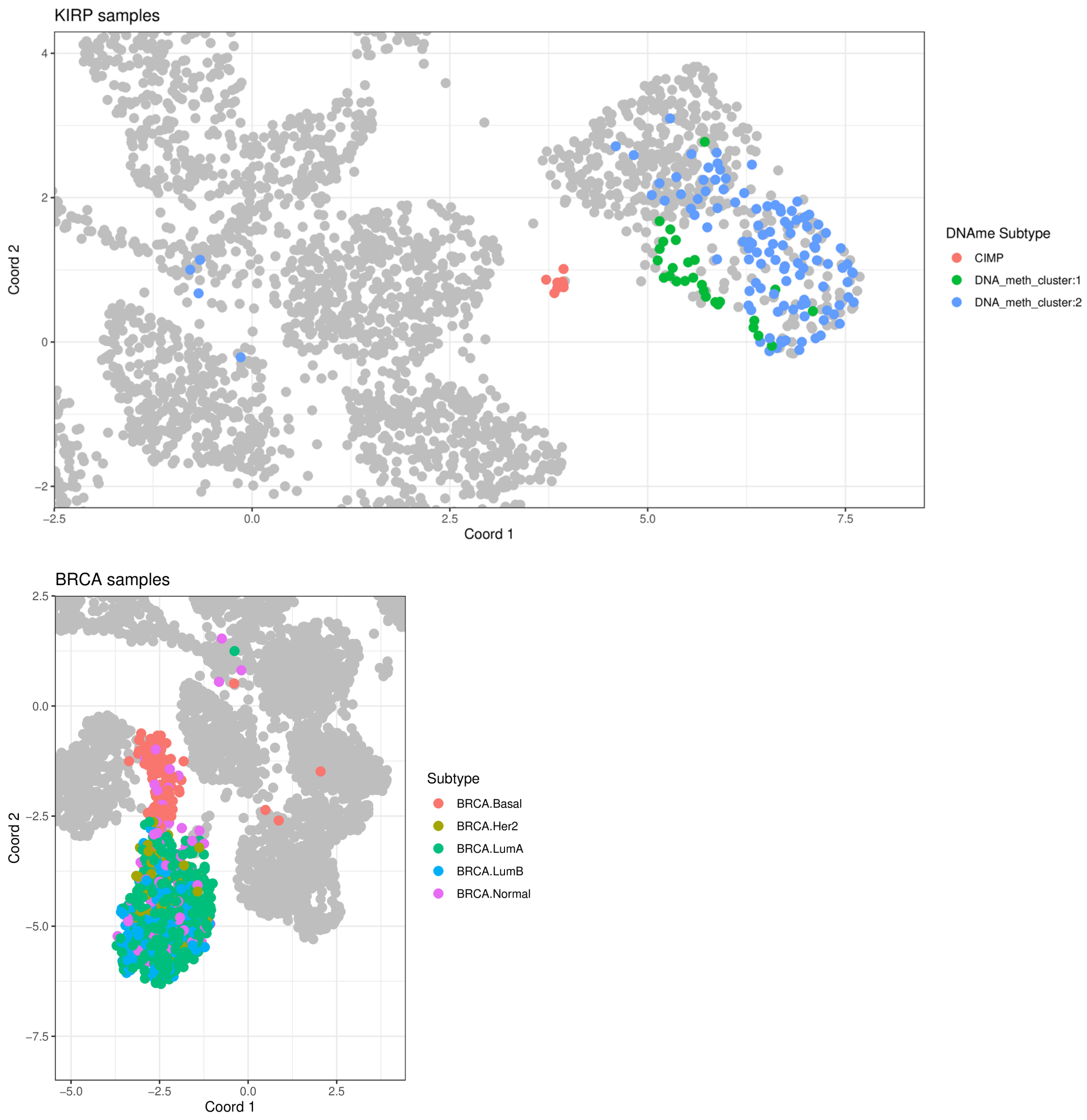

Supplementary Figure 15. UMAP projection for TCGA-KIRP(top) and TCGA-BRCA(bottom) samples by subtypes as in Figure 3D. Grey dots represent samples of other tumor types.

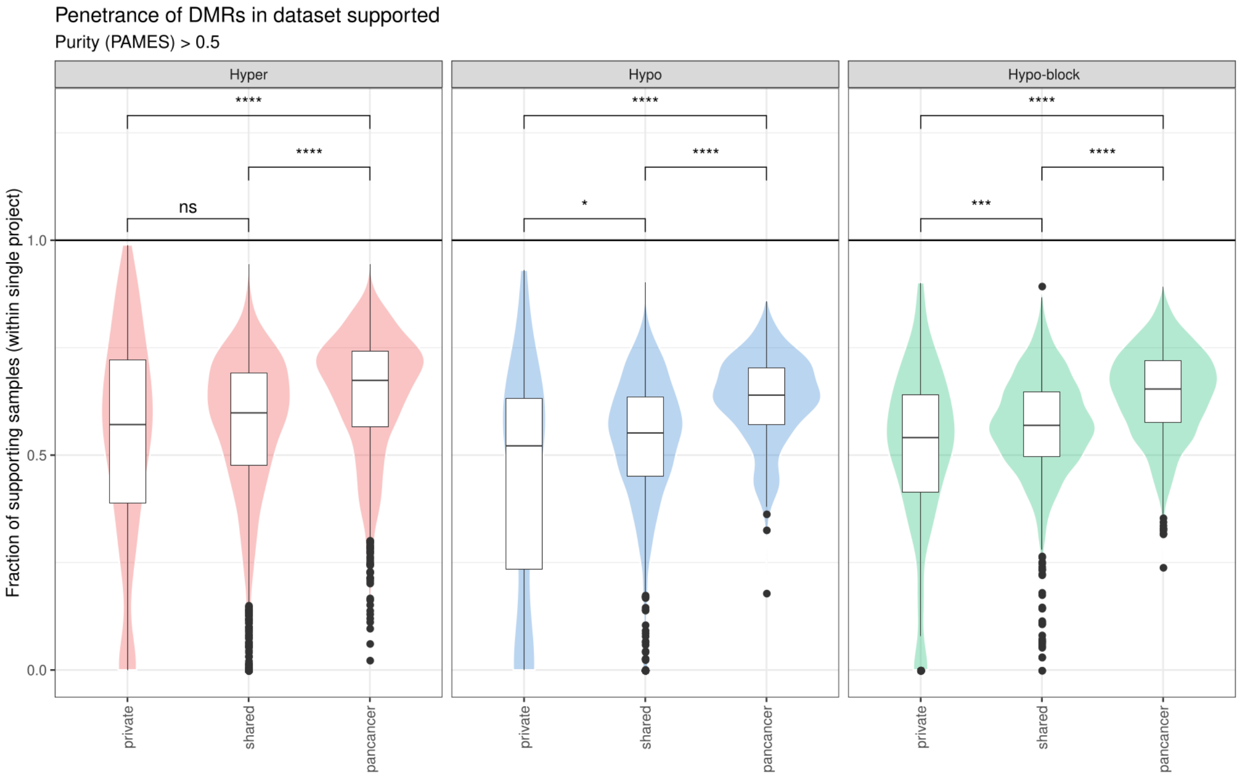

Supplementary Figure 16. Per DMR fraction of supporting samples. DMRs obtained from the comprehensive consensus have been divided into private (n = 1), shared (1<n<=7) and pancancer (n>7) based on the number of projects supporting the differential methylation. For each DMR, we measured the fraction of samples with differential methylation (z-score > 3 for hyper, z-score < -3 for hypo and hypo-blocks), considering only the tumor types in which the DMR is supported. Statistical significance is assessed using a two tailed WMW test. (ns: not significant, *:pval<.05, **:pval<.01, ***:pval<0.001, ****:pval<0.0001). Samples with purity lower than 50% have been excluded from this analysis.

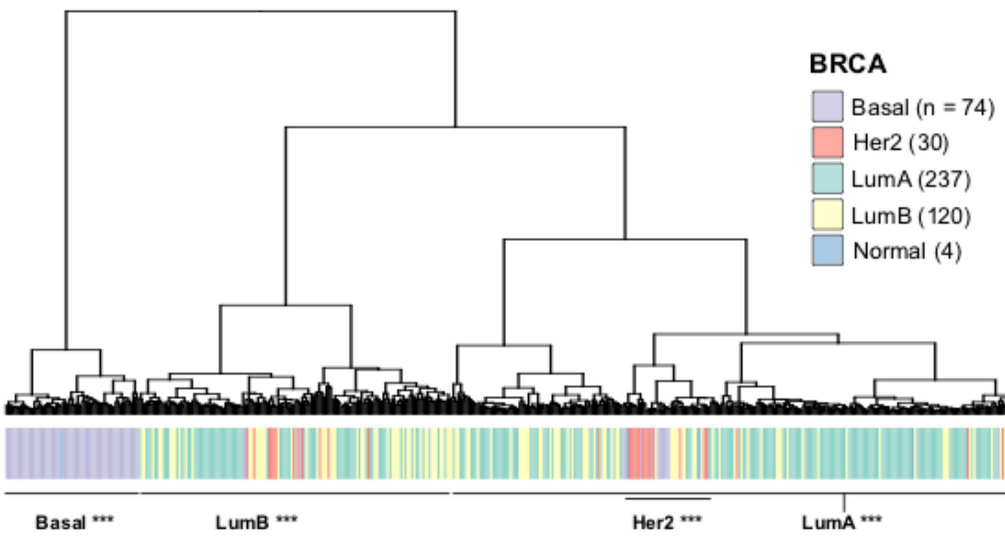

Supplementary Figure 17. Dendrogram showing the Ward's hierarchical clustering of hyper-DMRs in BRCA dataset, using '1-Pearson's Correlation Coefficient' as distance measure on averaged beta values. Annotation track reports the different molecular subtypes defined through the PAM50 signature. P-values were calculated by hyper-geometric test. . *** p-value < 0.001.

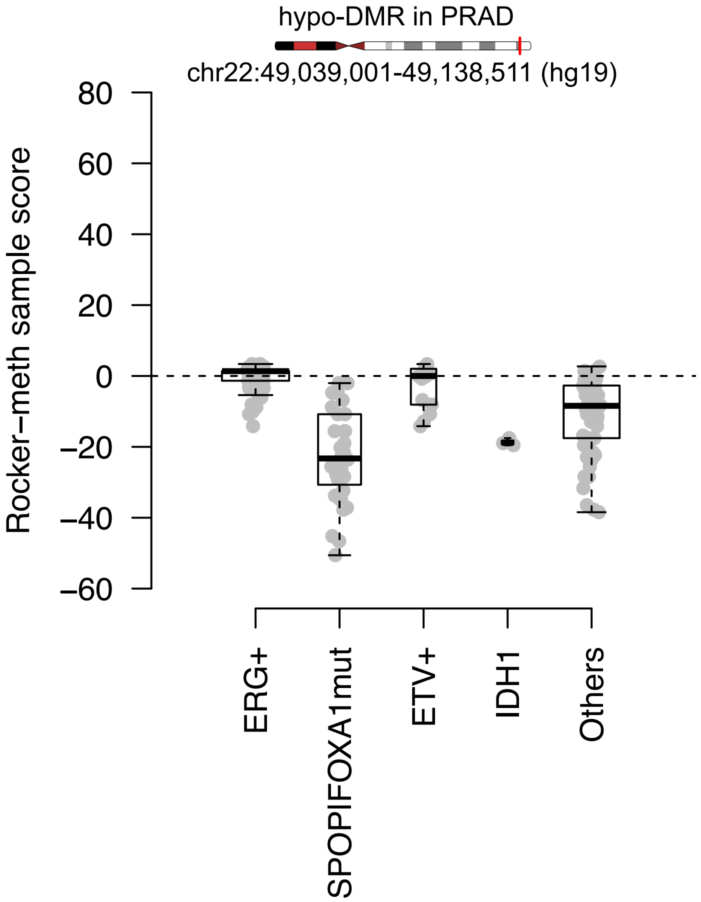

Supplementary Figure 18. Box plot showing the distribution of Rocker-meth sample score for a DMR mapping to 22q13.32 across the molecular subtypes of PRAD.

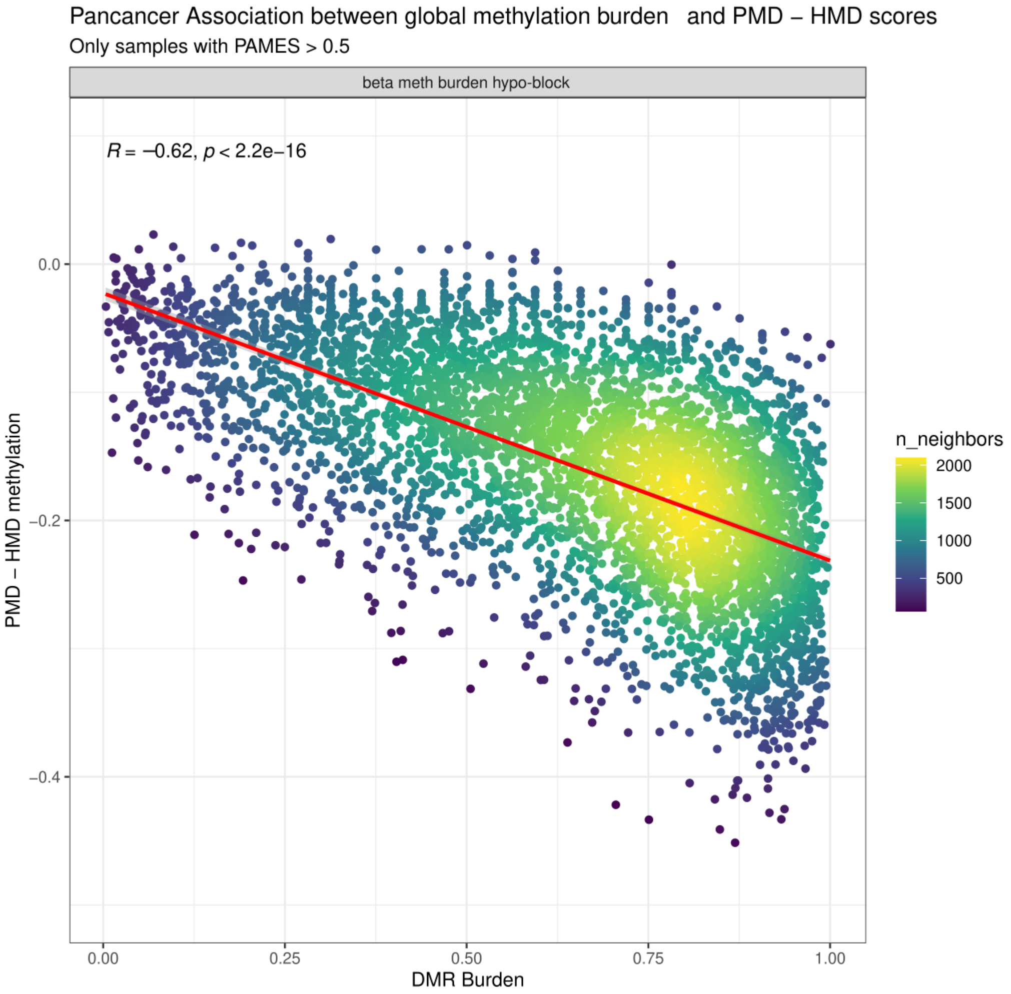

Supplementary Figure 19. Pancancer association between hypo-block burden (PSFSE for hypo-block DMRs) and PMD-HMD score (from Zhout et al). The color scale represents the point density. Samples with purity lower than 50% have been excluded from this analysis.

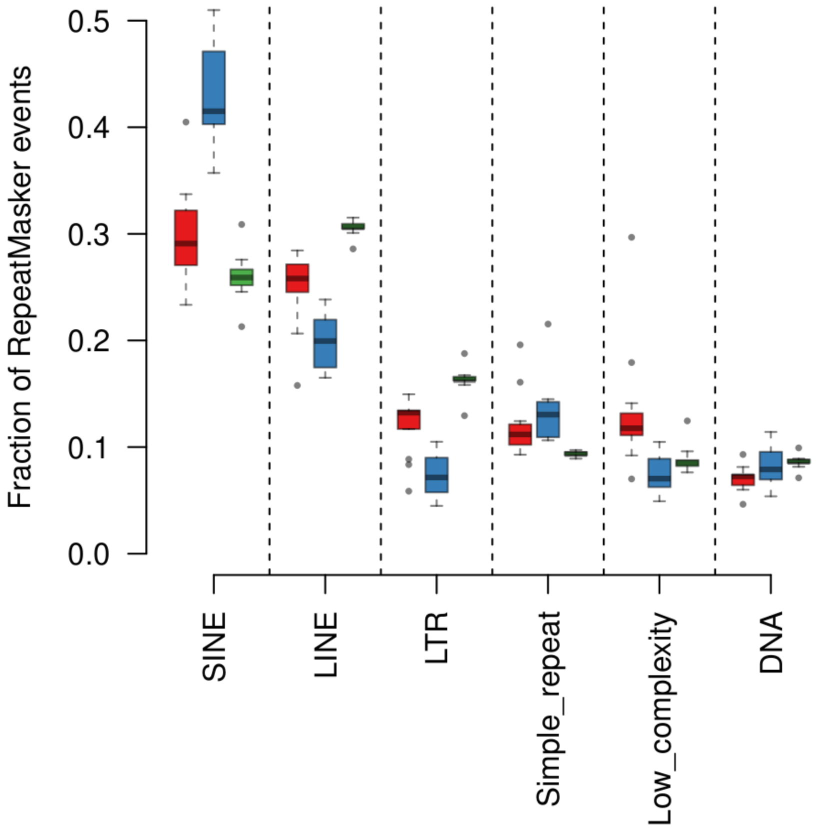

Supplementary Figure 20. Box plot showing the distribution of the fraction of RepeatMasker events overlapping hyper (red), hypo (blue) and hypo-block (green) DMRs across the 13 tumor types (THCA was excluded due to the absence of detected DMRs).

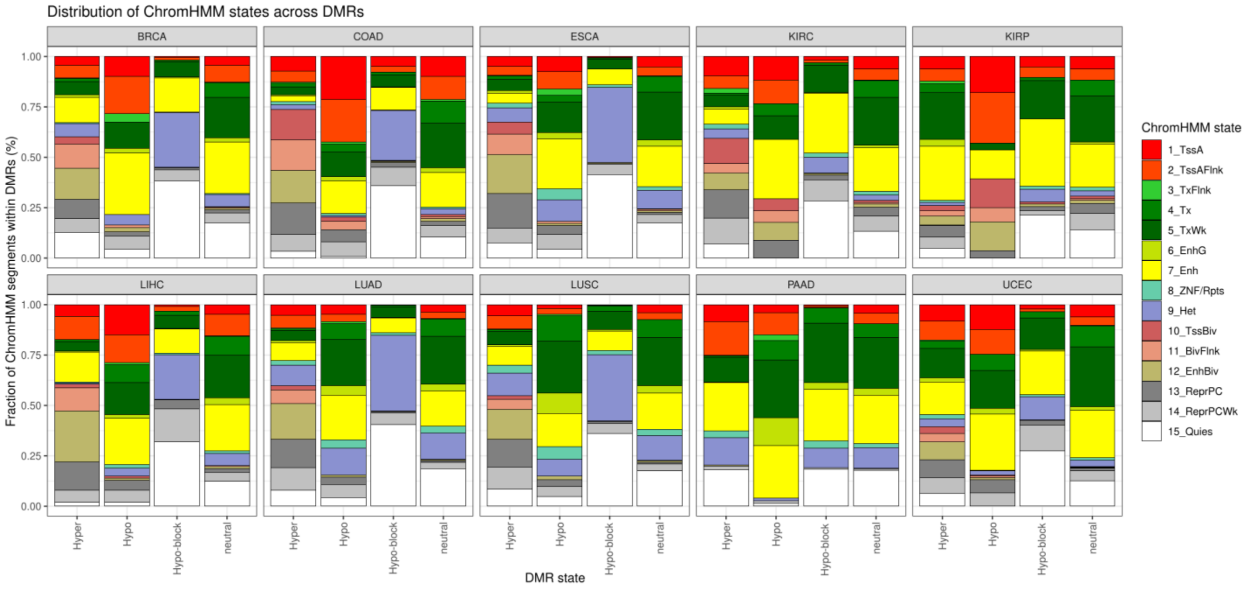

Supplementary Figure 21. Distribution of matched ChromHMM chromatin states within DMR classes and neutral segments obtained from Rocker-meth segmentation.

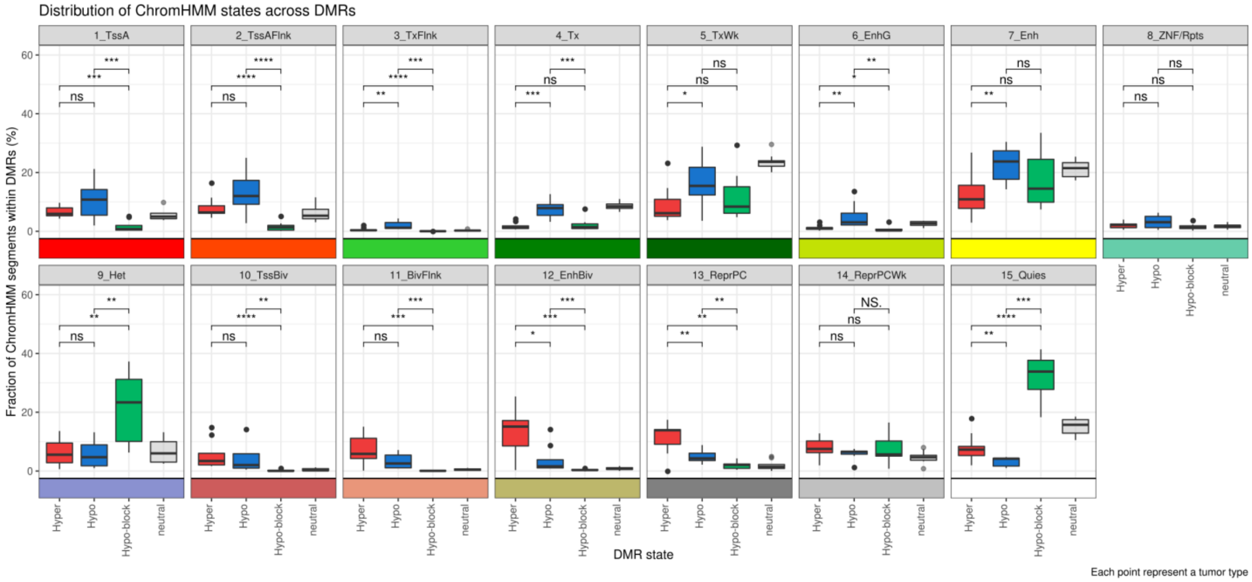

Supplementary Figure 22. Boxplot of all ChromHMM states from matching normal tissues (as in Figure 4C). Each dot represents a tumor type, and the fraction of segments within each class of DMRs is reported. Neutral is the non-differentially methylated genome obtained from RockerMeth segmentation. Statistical significance is assessed with pairwise Wilcoxon test.

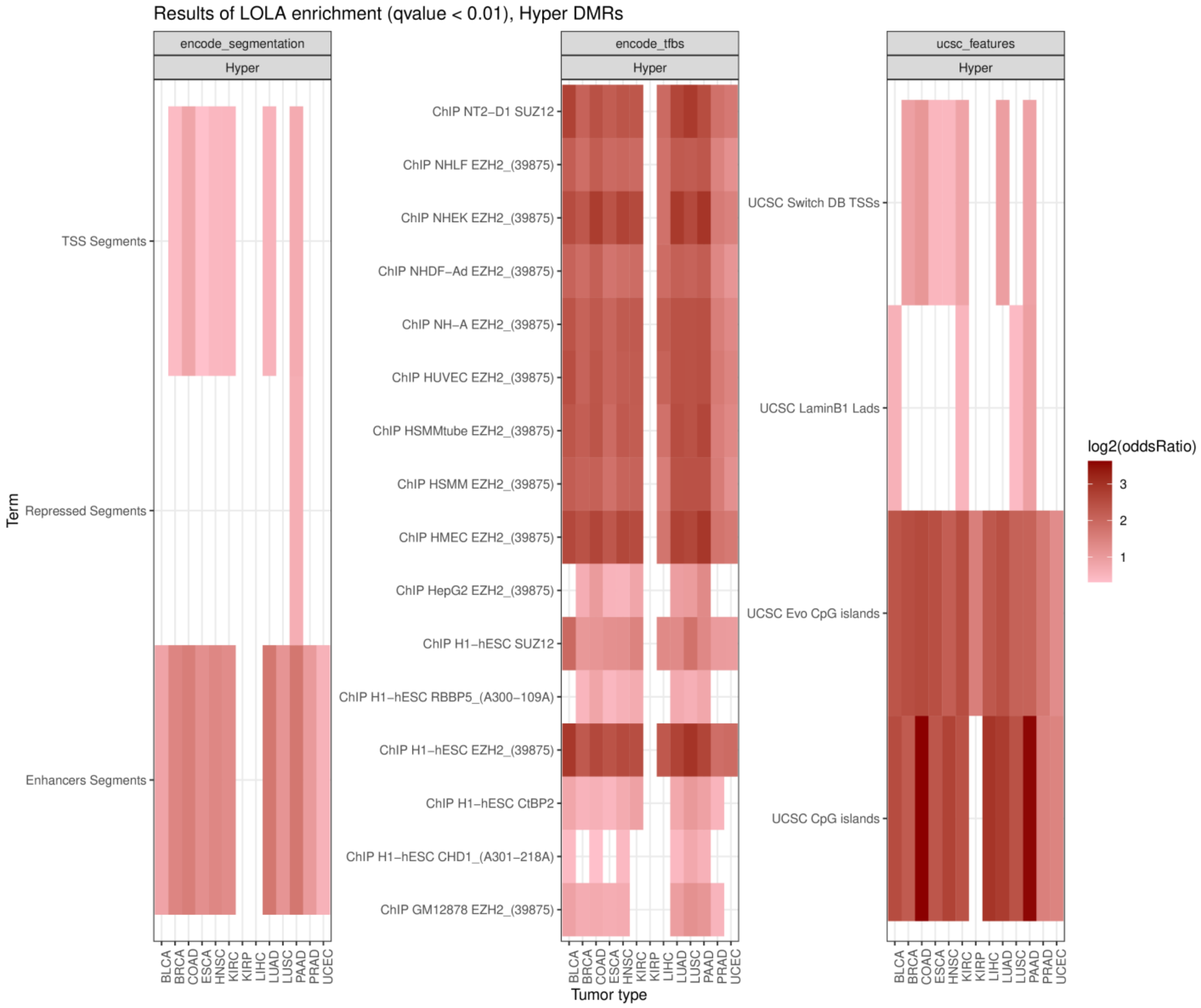

Supplementary Figure 23. Results of LOLA region enrichment analysis for Hyper DMRs obtained from HM450 TGCA data. Color scale captures the log2(OddsRatio) of enrichment. Reported terms are significant at FDR < 0.05.

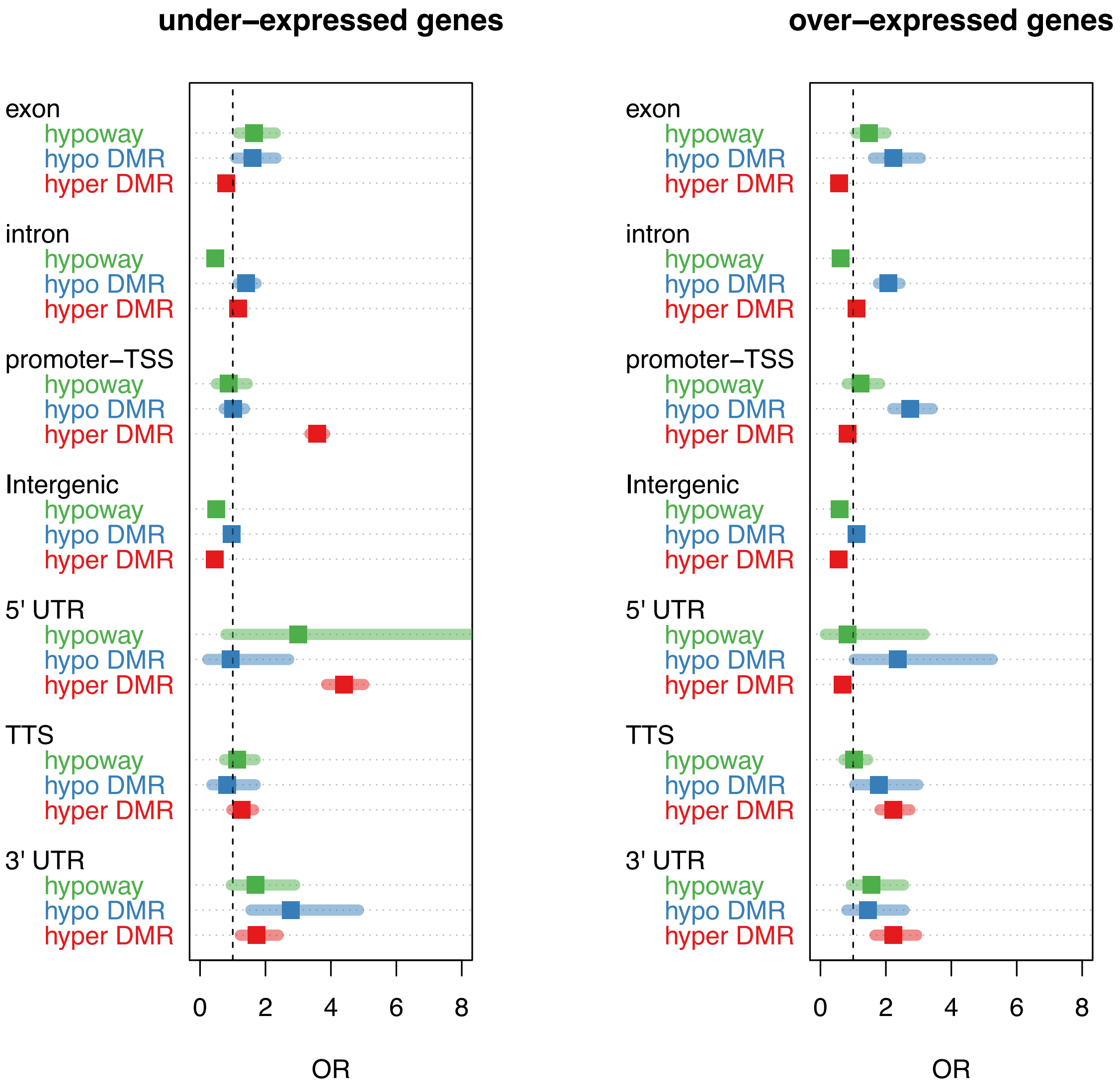

Supplementary Figure 24. Dot plot showing the Odds-ratio of the pan-cancer enrichment of hyper (red) and hypo (blue) Differentially Methylated Regions (DMRs) in under-expressed (left) and over-expressed (right) genes estimated by Fisher Exact Test (FET) for different genic annotations. Error bars show the 95% confidence interval.

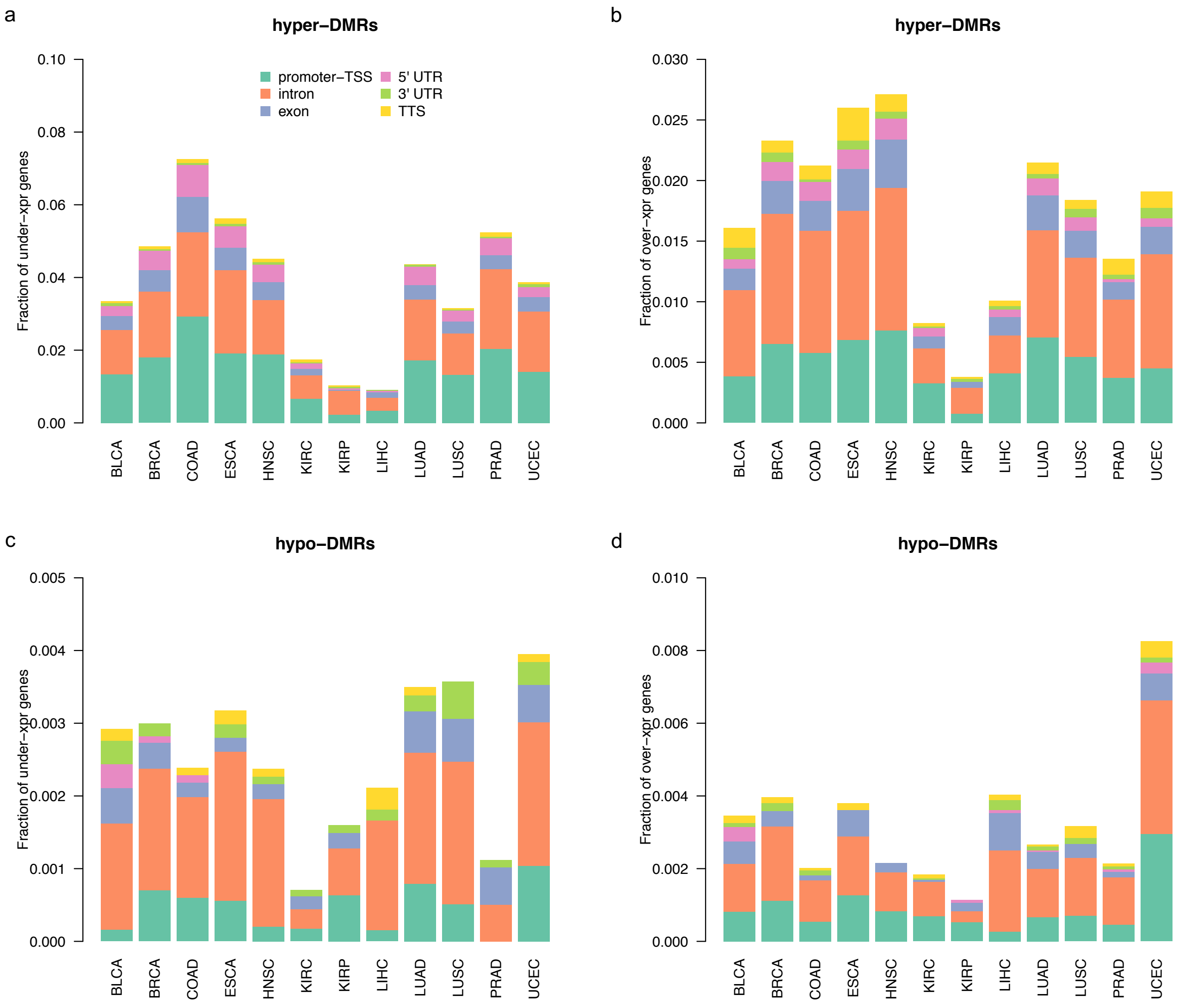

Supplementary Figure 25. Bar plots showing the fraction of differentially methylated and expressed genes by genic features and across 12 tumor types for a) hyper-methylated and under-expressed genes, b) hyper-methylated and over-expressed genes, c) hypo-methylated and under-expressed genes and d) hypo-methylated and over-expressed genes.

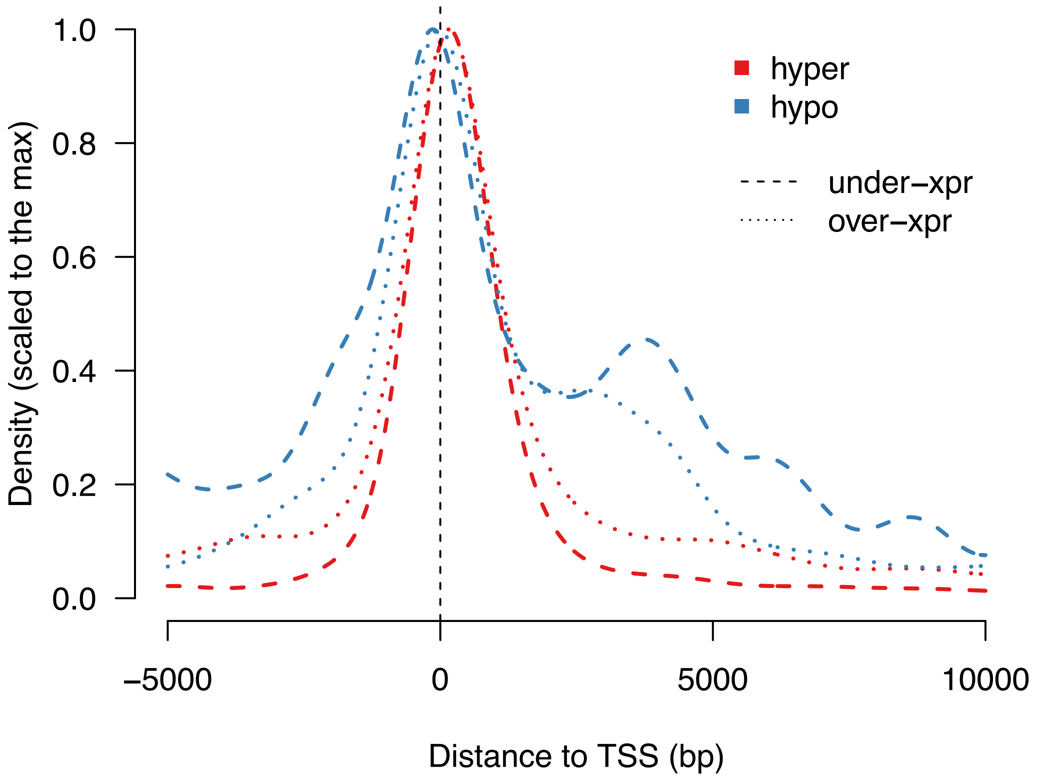

Supplementary Figure 26. Density plots of the distributions of DMR position (middle point) relative to the gene bodies for hyper (red) and hypo (blue) DMRs grouped by under-expressed (dashed) and over-expressed (dotted) genes.

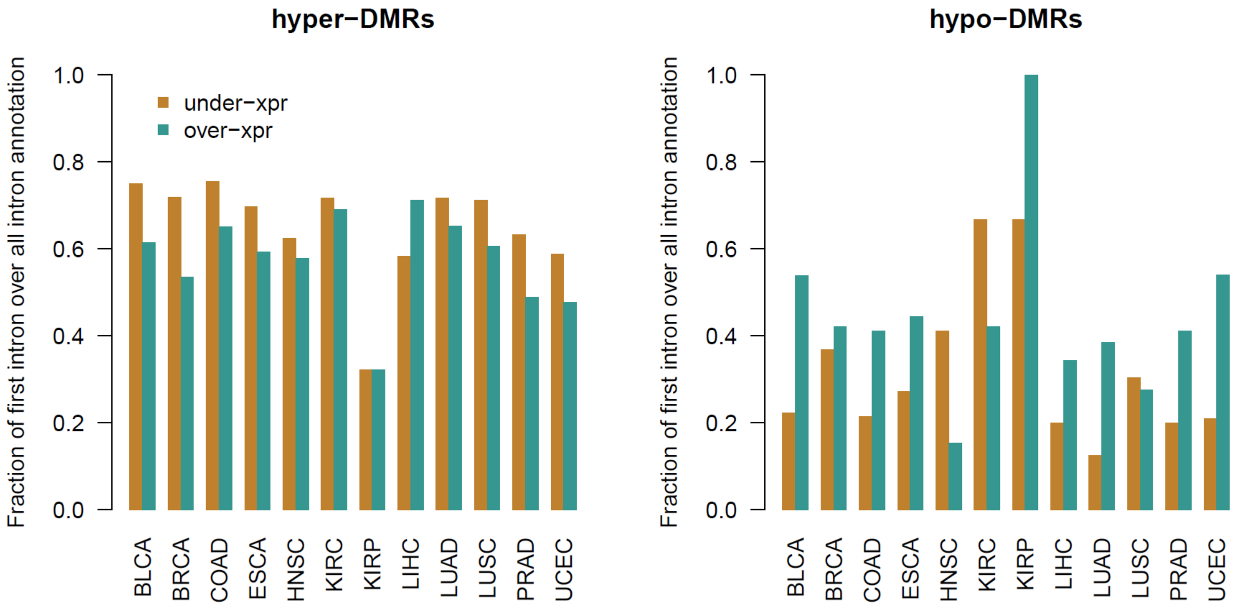

Supplementary Figure 27. Bar plots showing the fraction of DMRs annotated as "first intron" compared to all intronic DMRs. Brown: DMRs associated with under-expressed genes. Green: DMRs associated with over-expressed genes.

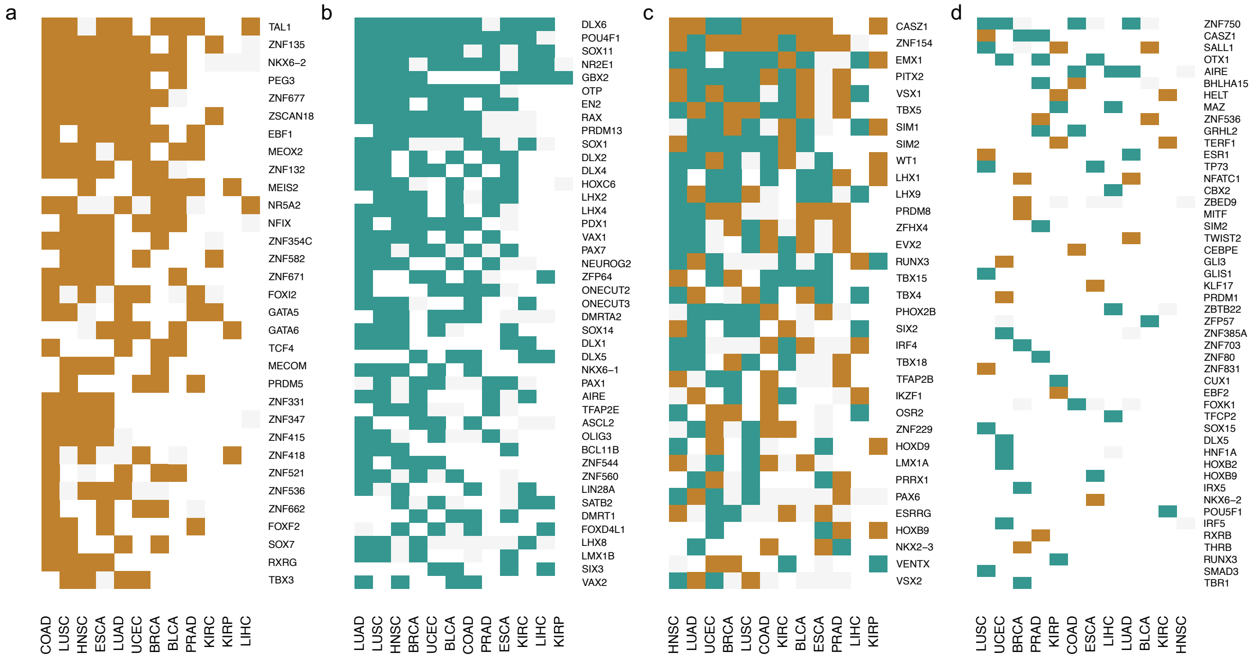

Supplementary Figure 28. Heat map of under-expressed (brown) and over-expressed (green) Transcription Factors (TFs) associated with hyper-DMRs and hypo-DMRs mapping to or close to their gene body. a) hyper-methylated and under-expressed TFs in at least 4 out of 12 tumor types and never over-expressed. b) hyper-methylated and over-expressed genes in 4 out of 12 tumor types and never under-expressed. c) hyper-methylated TFs being under-expressed and over-expressed in at least 2 tumor types. d) hypo-methylated and differentially expressed TFs.

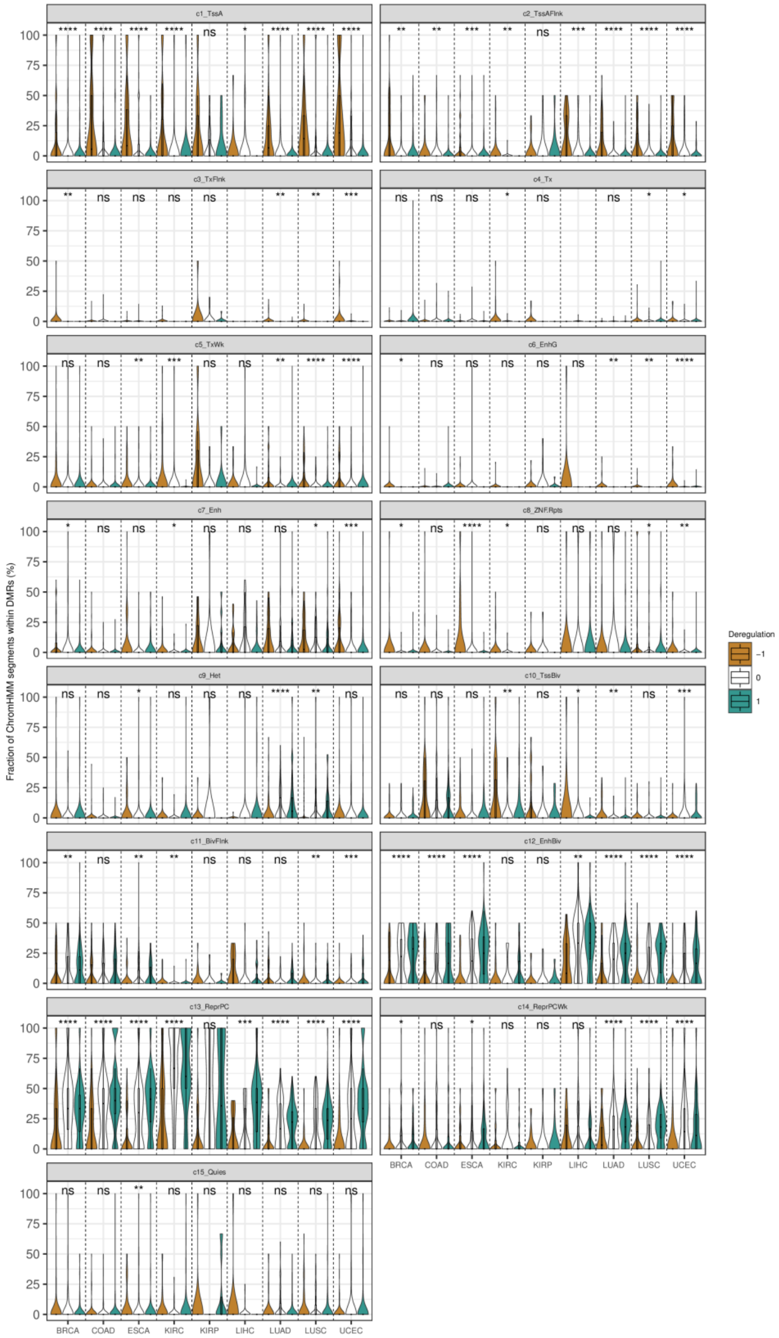

Supplementary Figure 29. Fraction of all matched ChromHMM states (ad in Figure 4F) in DMRs associated with transcription factors, grouped by deregulation. -1: TF is downregulated, 0: TF is not deregulated. 1 TF is upregulated (FDR < .05 is used to call differential expression).

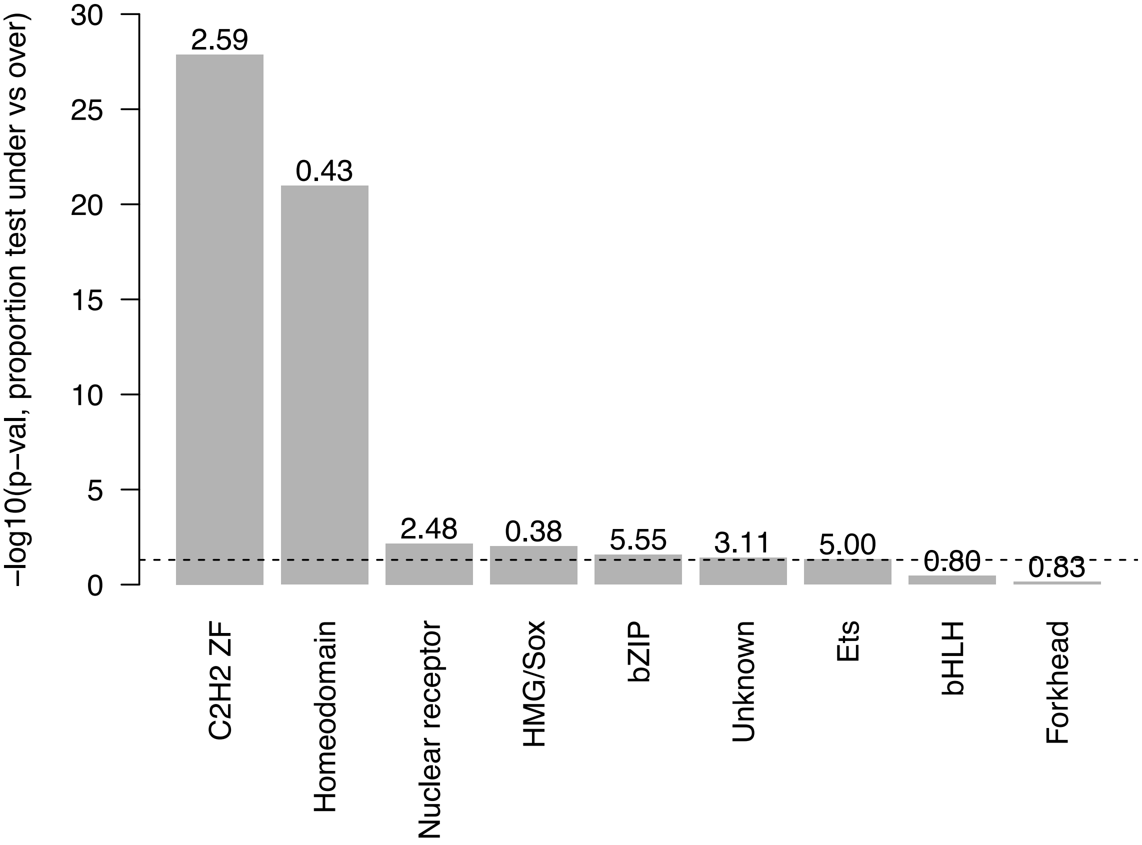

Supplementary Figure 30. Bar plot showing the –log10 of the p-values of the enrichment/depletion of the different TF classes in the set of under-expressed vs over-expressed TFs.

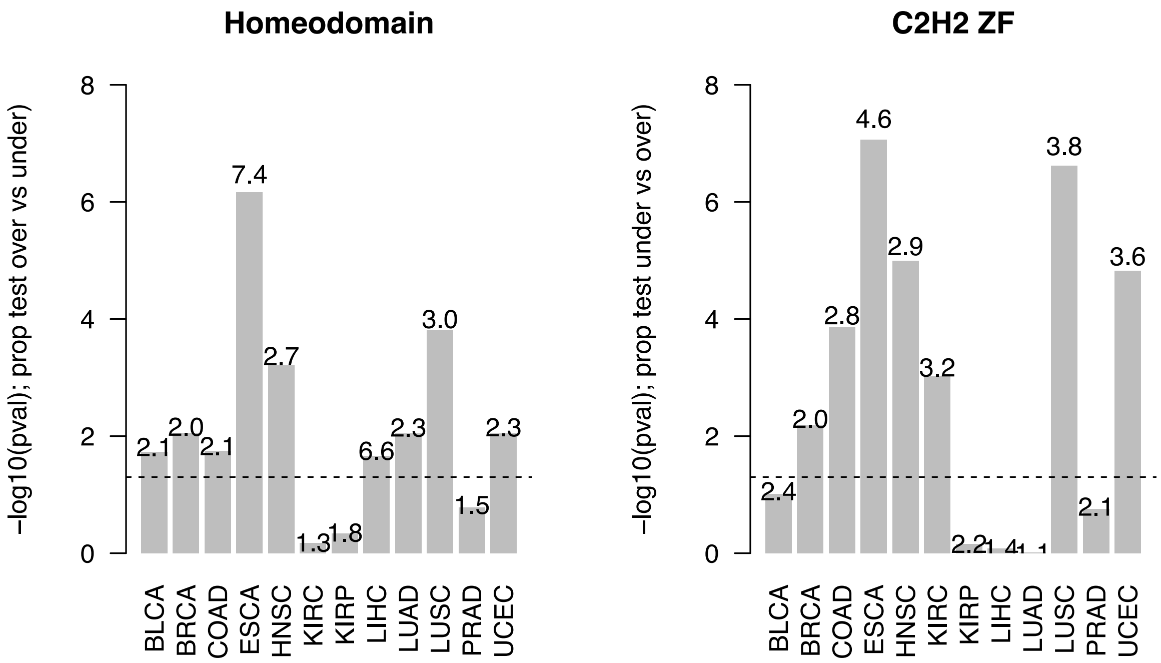

Supplementary Figure 31. Bar plot showing the –log10 of the p-values of the enrichment of the different TF classes across the 12 tumor types in the set of (left) over-expressed vs under-expressed TFs for the class homeodomain and (right) under-expressed vs over-expressed TFs for the class C2H2 ZF.

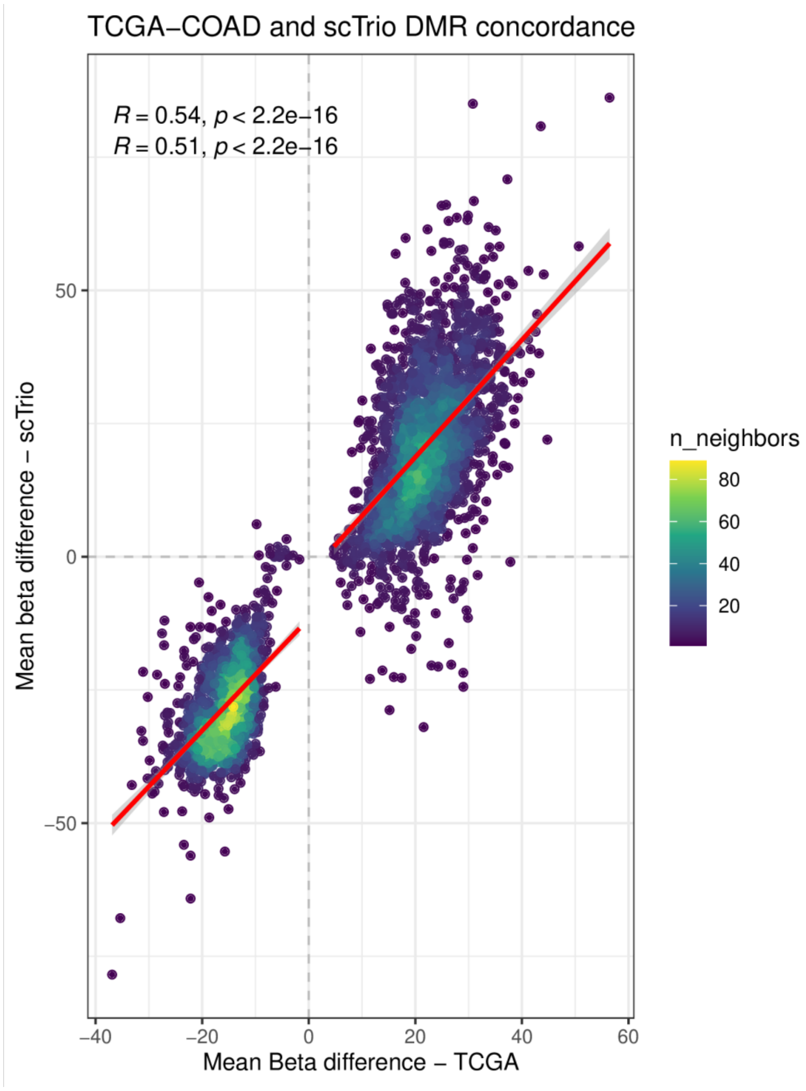

Supplementary Figure 32. Comparison of average beta difference between TCGA-COAD (bulk, HM450) and scTrio (single cell, WGBS). The set of DMRs nominated from TCGA-COAD has been used to collapse methylation values of single cells in average values and aggregated primary cells versus normal cells have been used to assess the average beta difference creating a pseudo-bulk across patients.

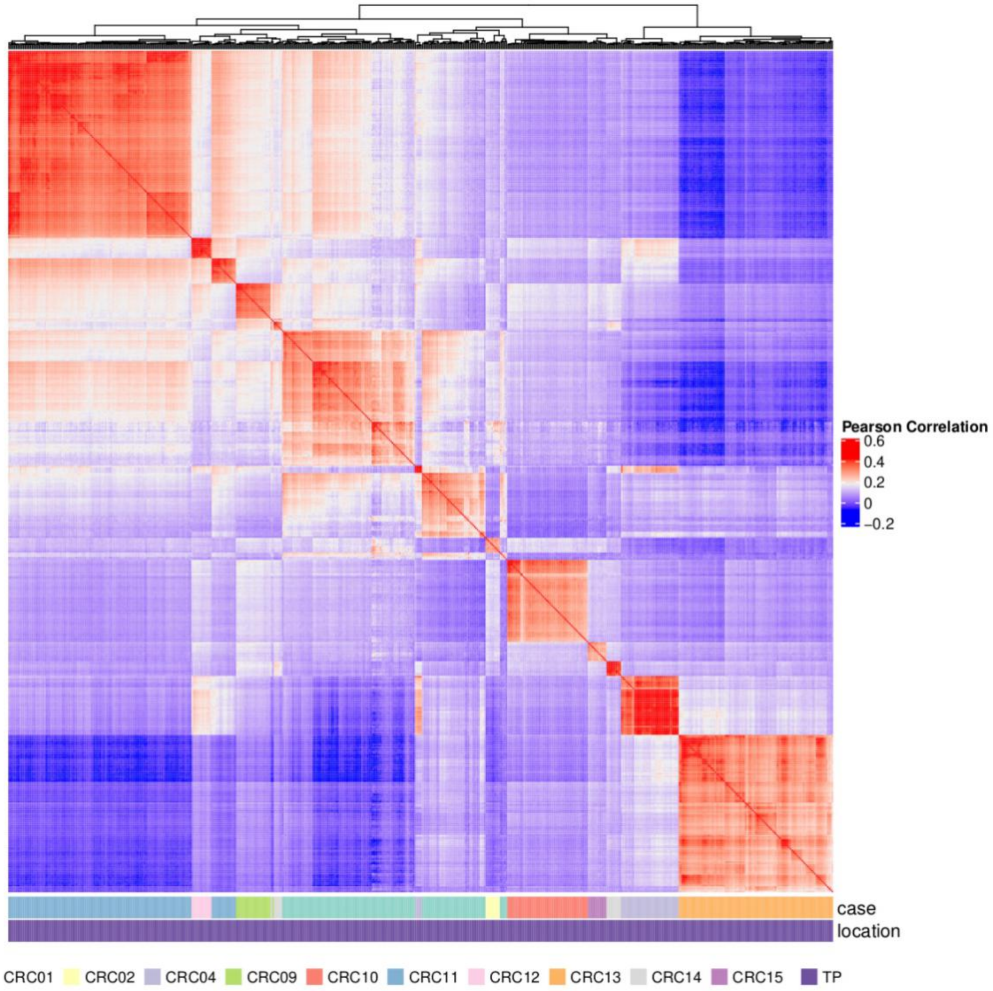

Supplementary Figure 33. Heatmap of Pearson correlation coefficients across tumor primary cells in scTrio dataset. The top 10% of the most variable tiles with less than 20% of missing values were used. Clustering was performed on Euclidean distance.

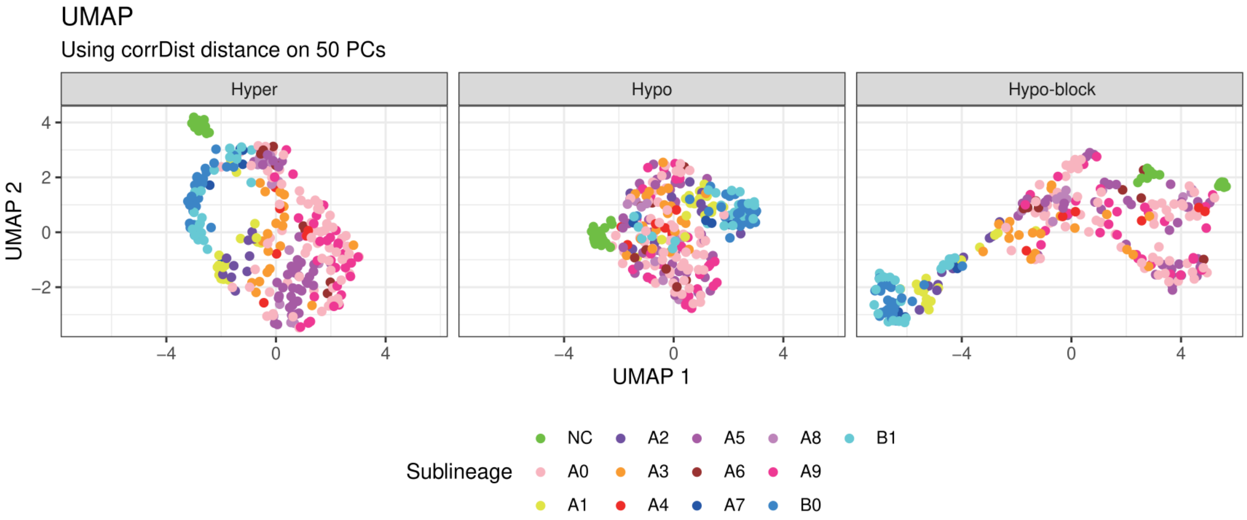

Supplementary Figure 34. UMAP of scTrio cells from CRC01 case. The coordinates are obtained as in Figure 5D but based only on Beta values in single DMR sets from the TCGA-COAD catalog.

a

b

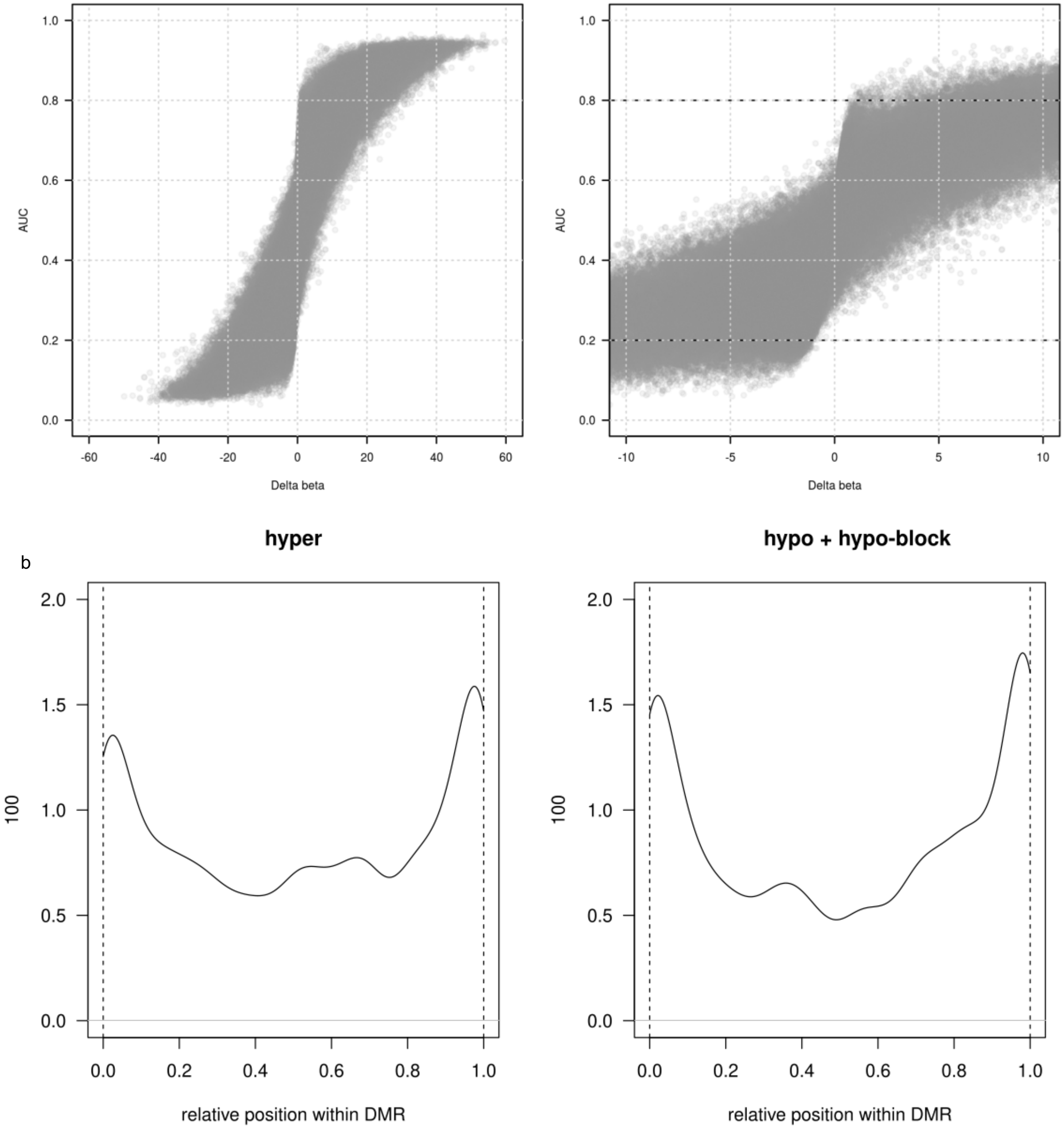

Supplementary Figure 35. a) Scatter plot of AUC versus delta beta (mean methylation difference between tumor and control samples) in the TCGA-PRAD dataset. A magnification of the region |delta beta| < 10 is reported on the right panel. b) Density plot of the relative position within DMRs for left) sites having AUC > 0.8 and 0≤delta beta < 10 and right) sites having AUC < 0.2 and -10 < delta beta ≤ 0. Relative position is calculated as the difference between site position and DMR start then divided by DMR length.

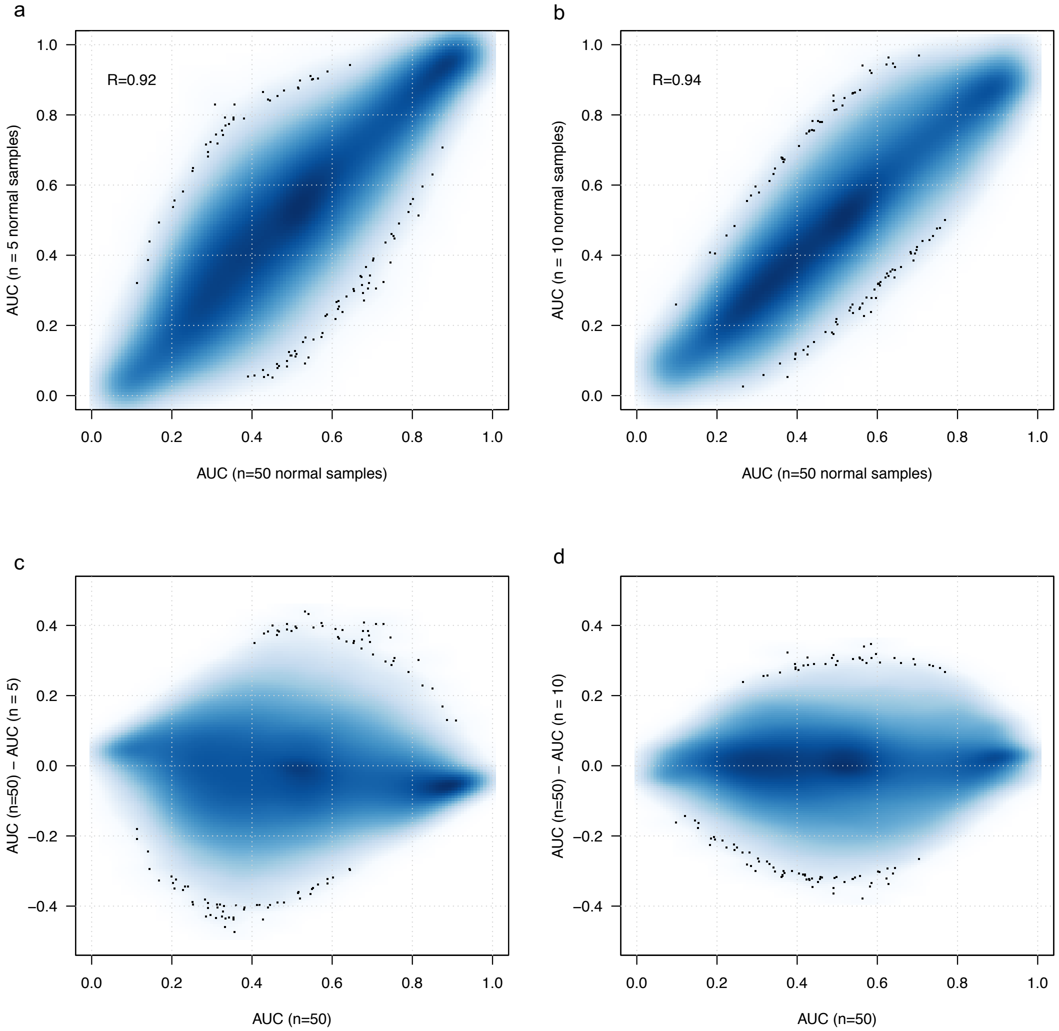

Supplementary Figure 36. a) Scatter plot of AUCs obtained from differential methylation analysis of TCGA-PRAD tumor samples (n=498) using 5 normal samples (y-axis) versus all normal samples (n=50). b) Scatter plot of AUCs obtained from differential methylation analysis of TCGA-PRAD tumor samples (n=498) using 10 normal samples (y-axis) versus all normal samples c) Scatter plot of the difference between AUCs using all normal samples and 5 normal samples versus AUCs using all normal samples. d) Scatter plot of the difference between AUCs using all normal samples and 10 normal samples versus AUCs using all normal samples. For the two subsets of normal samples, values were estimated by averaging AUCs from 20 random samplings.

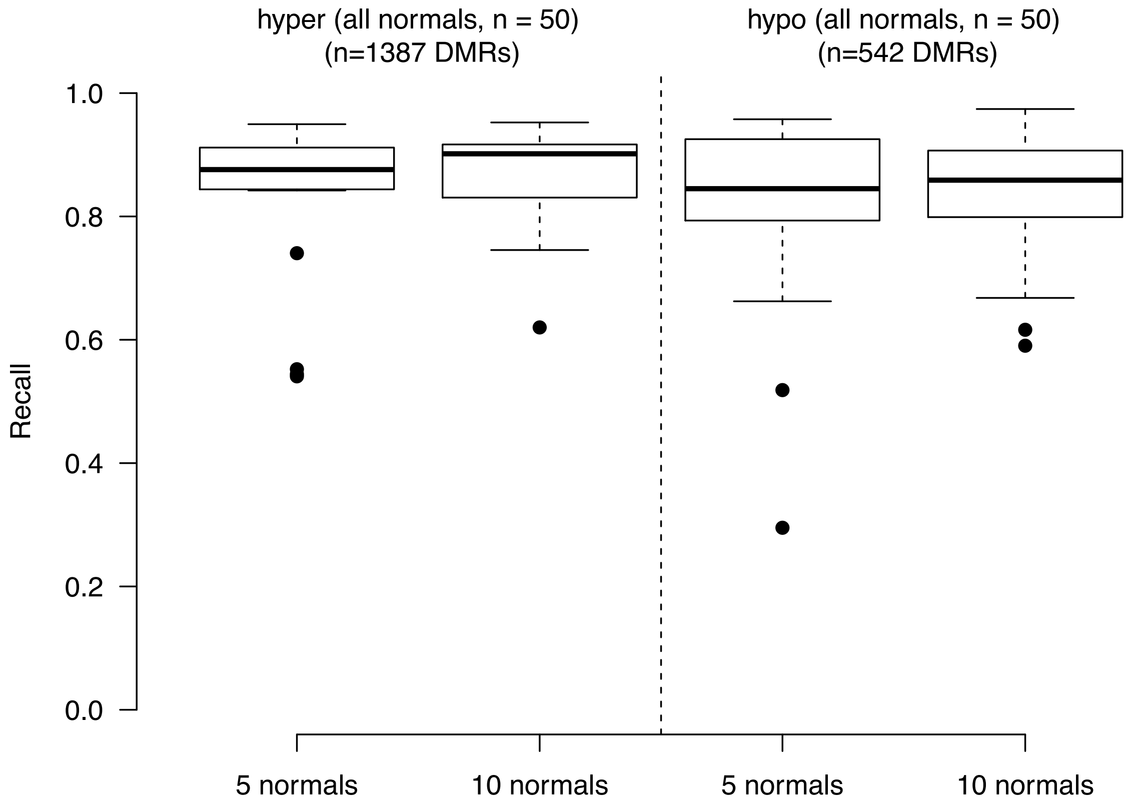

Supplementary Figure 37. Box plot of the distribution of the fraction of true (i.e., using all normal samples) DMRs detected by Rocker-meth using 5 and 10 normal samples for (left) hyper and (right) hypo events. Data are from 20 samplings.

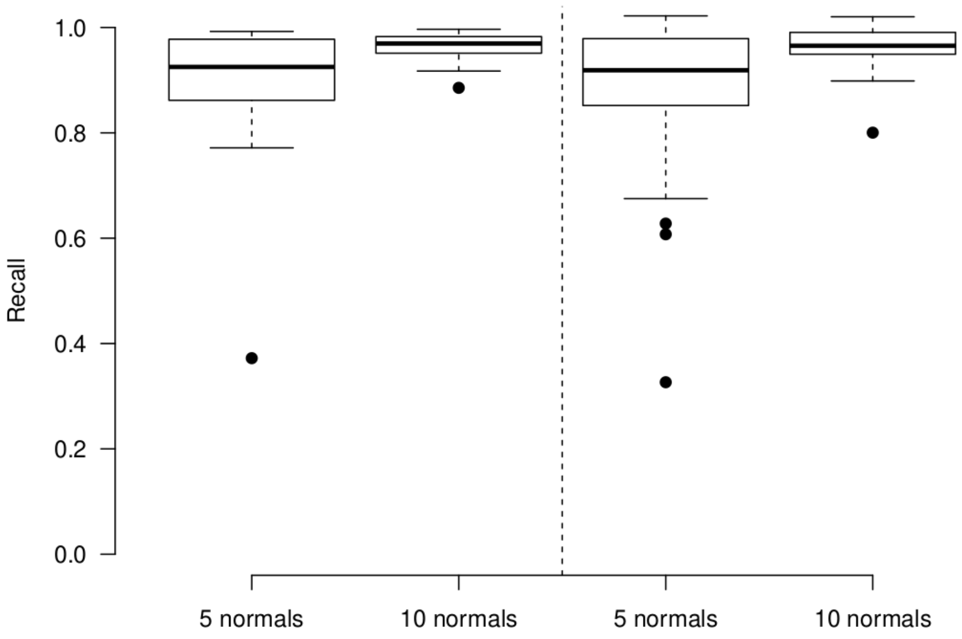

Supplementary Figure 38. Box plot of the distribution of the fraction of true (i.e., using all normal samples) DMRs detected by Rocker-meth using 5 and 10 normal samples for (left) hyper and (right) hypo events. Data are from 20 samplings and DMRs are not filtered for FDR.

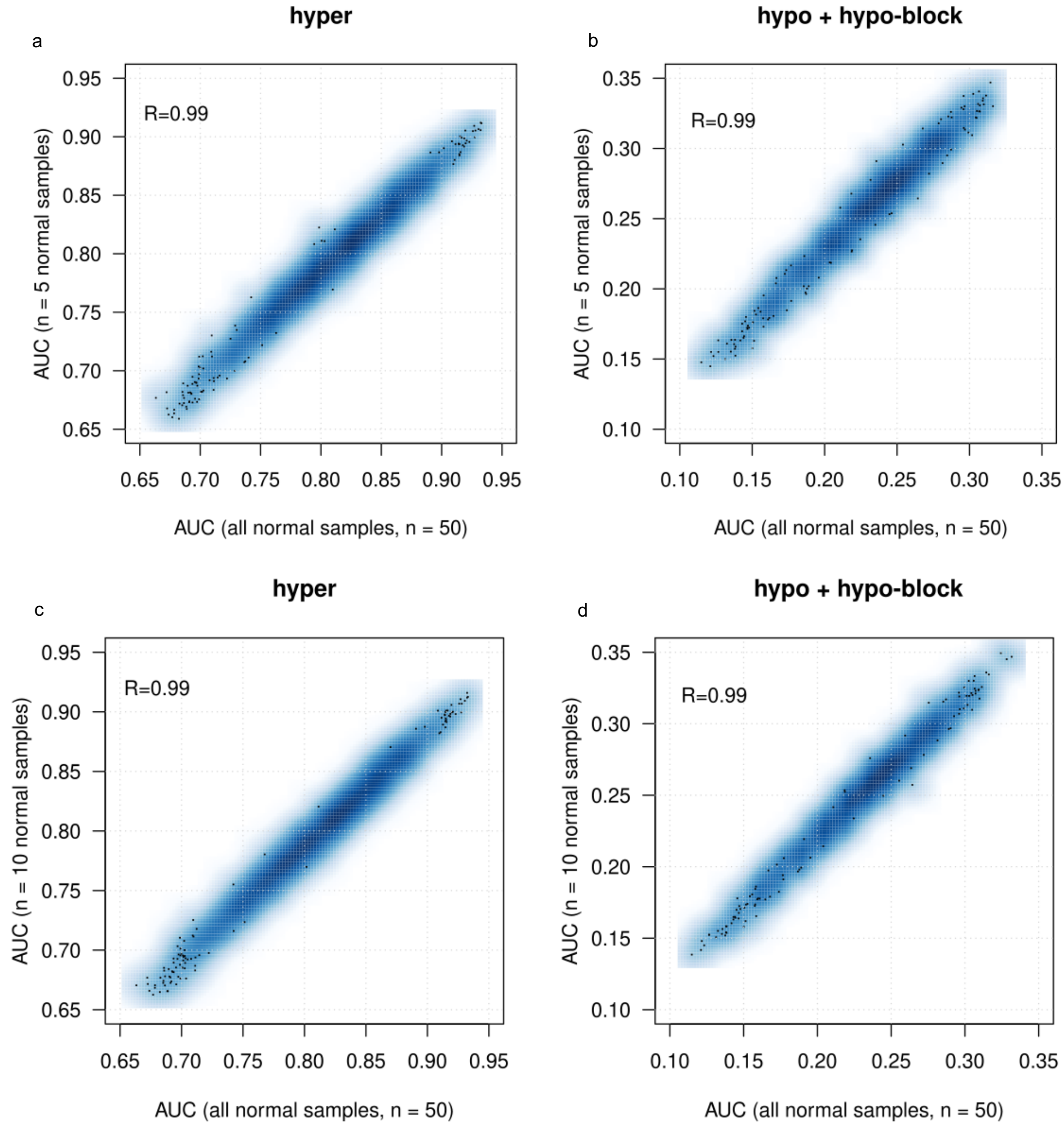

Supplementary Figure 39. a) Scatter plot of AUCs obtained from differential methylation analysis of TCGA-PRAD tumor samples (n=498) using 5 normal samples (y-axis) versus all normal samples (n=50). b) Scatter plot of AUCs obtained from differential methylation analysis of TCGA-PRAD tumor samples (n=498) using 10 normal samples (y-axis) versus all normal samples c) Scatter plot of the difference between AUCs using all normal samples and 5 normal samples versus AUCs using all normal samples. d) Scatter plot of the difference between AUCs using all normal samples and 10 normal samples versus AUCs using all normal samples. For the two subsets of normal samples, values were estimated by averaging AUCs from 20 random samplings. Data refers to AUCs in the DMRs detected by Rocker-meth in the TCGA-PRAD dataset.

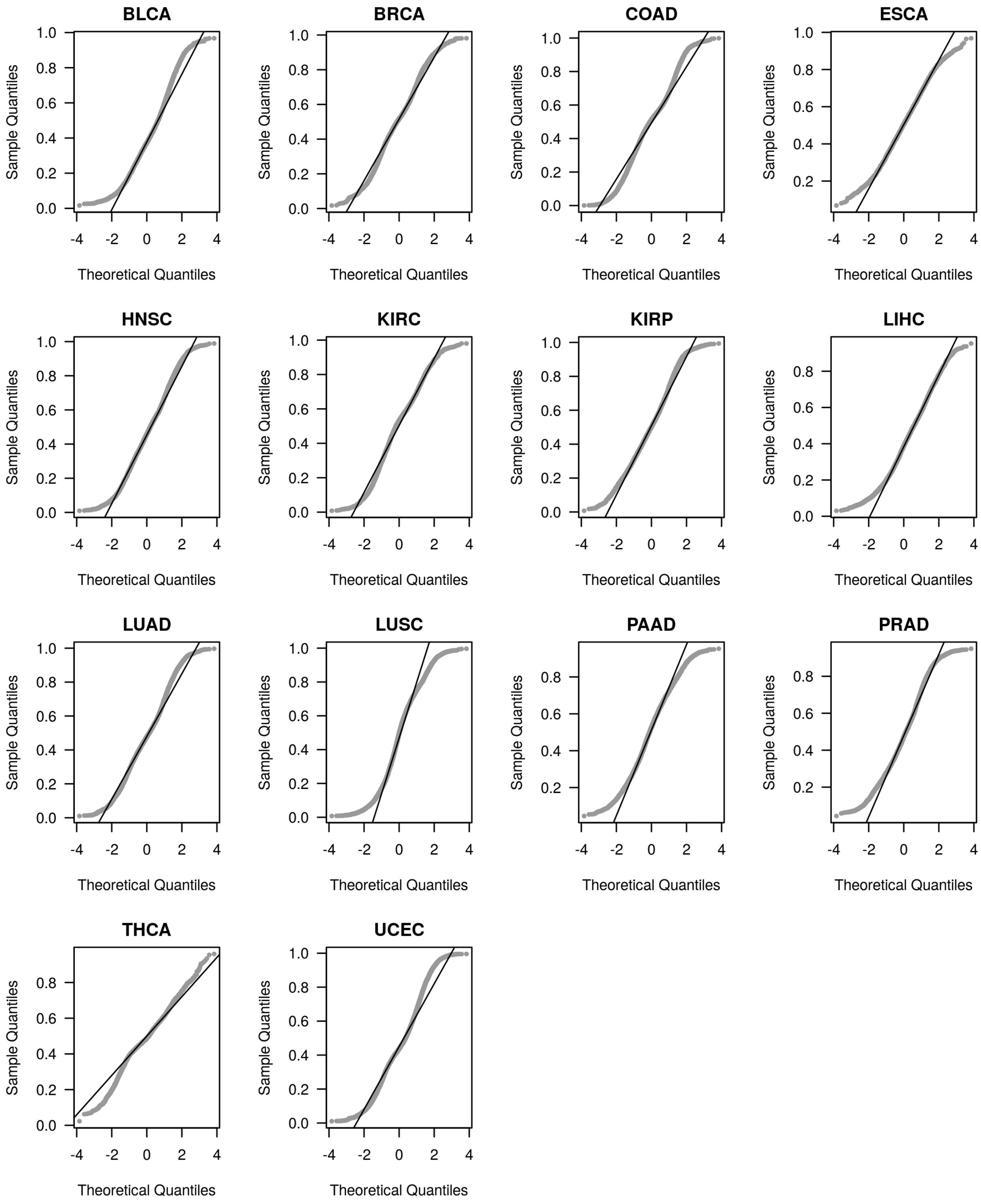

Supplementary Figure 40. Normal qq plots of the AUC scores across the 14 TCGA datasets considered in this study. For each dataset, n=104 AUC scores were randomly sampled.
