# Supplementary material for: Charting differentially methylated regions in cancer with Rocker-meth
Source: Commun Biol. 2021 Nov 2;4:1249. doi: 10.1038/s42003-021-02761-3 (PMC8563962; doi:10.1038/s42003-021-02761-3)
Supplement: Supplementary file 3 — Description of Additional Supplementary Files [file 42003_2021_2761_MOESM3_ESM.pdf]

Description of Additional Supplementary Files

File name: Supplementary Data 1-11
Description:

Supplementary Data 1: Characteristics of artificial datasets.

Supplementary Data 2: Statistical evaluation of Rocker-meth and other state-of-the-art methods on WGBS artificial datasets.

Supplementary Data 3: Statistical evaluation of Rocker-meth and other state-of-the-art methods on RRBS artificial datasets.

Supplementary Data 4: Statistical evaluation of Rocker-meth and other state-of-the-art methods on 450K artificial datasets.

Supplementary Data 5: Catalog of DMRs across 14 tumor types from 450K data. Coordinates are hg19 based.

Supplementary Data 6: Refined catalog of DMRs across 14 TCGA tumor types. Coordinates are hg19 based.

Supplementary Data 7: Fraction of DMRs in different genomic features across 13 TCGA datasets.

Supplementary Data 8: -log10 of the p-values of the enrichment analysis (by Fisher Exact Test) of differentially expressed genes and differentially methylated regions across tumor types.

Supplementary Data 9: Details about DNA methylation TCGA dataset.

Supplementary Data 10: Details about RNA-seq TCGA dataset.

Supplementary Data 11: Summary of genomic features by HOMER annotation in the illumina infinium human methylation 450k beadchip.
